# Fast life history traits promote invasion success in amphibians and reptiles

**DOI:** 10.1111/ele.12728

**Published:** 2017-01-04

**Authors:** William L. Allen, Sally E. Street, Isabella Capellini

**Affiliations:** ^1^ School of Environmental Sciences University of Hull Cottingham Road Hull HU6 7RX UK; ^2^ Department of Biosciences Swansea University Singleton Park Swansea SA2 8PP Wales UK

**Keywords:** Amphibians, biological invasions, comparative analyses, invasion biology, invasive species, life history theory, population dynamics, population growth, reptiles, transient dynamics

## Abstract

Competing theoretical models make different predictions on which life history strategies facilitate growth of small populations. While ‘fast’ strategies allow for rapid increase in population size and limit vulnerability to stochastic events, ‘slow’ strategies and bet‐hedging may reduce variance in vital rates in response to stochasticity. We test these predictions using biological invasions since founder alien populations start small, compiling the largest dataset yet of global herpetological introductions and life history traits. Using state‐of‐the‐art phylogenetic comparative methods, we show that successful invaders have fast traits, such as large and frequent clutches, at both establishment and spread stages. These results, together with recent findings in mammals and plants, support ‘fast advantage’ models and the importance of high potential population growth rate. Conversely, successful alien birds are bet‐hedgers. We propose that transient population dynamics and differences in longevity and behavioural flexibility can help reconcile apparently contrasting results across terrestrial vertebrate classes.

## Introduction

A key question in ecology and evolution is why some small populations thrive whilst others go extinct. The dynamics of small populations are at the heart of natural colonisations, radiations and changes in ecological communities, and are central in applied problems such as *in situ* species conservation and reintroduction. Biological invasions present an opportunity to address this question, as invasions generally begin with small populations, and have been recorded in sufficient frequency and detail to allow for investigating which factors lead to organisms succeeding outside their native range (Kolar & Lodge 2001; Sax *et al*. 2007) at each stage of the invasion pathway, from introduction through to establishment and spread (Blackburn *et al*. 2011). While it is well established that invasion success increases strongly with higher introduction effort (also called ‘propagule pressure’; Lockwood *et al*. 2005; Jeschke & Strayer 2006; Blackburn *et al*. 2015), and is facilitated by the similarity between the climates of native and introduced ranges (Kolar & Lodge 2001; Mahoney *et al*. 2015), there is still considerable interspecific variation in invasion success that remains unexplained. In this context it is debated whether some species are intrinsically better or worse invaders than others (Kolar & Lodge 2001; Hayes & Barry 2008; Sol & Maspons 2016). Life history traits, such as offspring/seed number, frequency of reproduction, age at sexual maturity and reproductive lifespan, are the most likely candidate traits underlying interspecific differences in introduction outcomes because they directly relate to vital rates (e.g. survival, reproduction) and determine how populations of a given species grow (Sæther *et al*. 2013). However, there is conflicting theory on exactly how life history traits influence growth rates of small populations and support range expansion. Although several studies include life history traits in analyses on invasions, only two studies to date explicitly test the predictions of alternative theoretical models of population growth in birds and mammals respectively (Sol *et al*. 2012; Capellini *et al*. 2015), reaching opposite conclusions. As a result, we still do not know to what extent there are general principles underpinning population growth from small sizes, or whether responses are taxon specific. Here, we investigate how life history traits support population growth as predicted by opposing theoretical models in alien reptiles and amphibians, the two phylogenetically closest vertebrate classes to mammals and birds, previously studied, and begin to draw general conclusions.

Classic models of population growth (Pimm 1991) predict an advantage for species with ‘fast’ life history traits, such as high fecundity and early maturity. These species have the potential to grow in population size rapidly, reducing the period during which they are at highest risk of going extinct due to stochastic events (Pimm 1991). The population growth rate potential for fast species should continue to be an advantage at the stage of spread, as populations increase from medium to large sizes, and fast traits may also support range expansion (Blackburn *et al*. 2015; Capellini *et al*. 2015). Alternative ‘slow advantage’ demographic models (Jeppsson & Forslund 2012; Sæther *et al*. 2013) predict that species with slower life history strategies are better able to establish because of several mechanisms. By prioritising survival over reproduction, species with slow life history strategies experience less demographic variability between years, which buffers populations against the consequences of negative stochastic events and the drastic population declines that can lead to extinction (Sæther *et al*. 2004, 2013; Jeppsson & Forslund 2012). ‘Slow’ species can also delay reproduction, and store the energy that would have been expended until conditions are more favourable (the ‘storage effect’, Cáceres 1997). Finally, bet‐hedging strategies that reduce temporal variation in fitness, for example by investing less into each reproductive attempt, might increase success in unpredictable environments, despite a short‐term cost of reduced mean individual fitness (Starrfelt & Kokko 2012). Bet‐hedging is generally associated with slow life history traits, especially long reproductive lifespan, but it can also be achieved by increasing the frequency of reproduction (Sol *et al*. 2012) or delaying germination (Venable 2007).

Empirical tests do not always identify life history traits as important factors in biological invasions (e.g. Hayes & Barry 2008; Liu *et al*. 2014), and when they do, there is limited consistency in which traits are important. Invasive conifers have early maturity and produce large numbers of small seeds frequently (Richardson & Rejmánek 2004), whereas successful mammals have large and frequent litters (Capellini *et al*. 2015). These results support fast advantage models of population growth. Slow advantage models are instead favoured in birds (Sol *et al*. 2012) as successfully established species have small clutches and divide lifetime reproduction out over multiple events. These traits should allow avian invaders to engage in bet‐hedging, buffering and storage strategies, that reduce the fitness consequences of reproductive failure and enable individuals to ‘wait out’ negative stochastic events (Starrfelt & Kokko 2012). Existing studies of amphibian and reptile invasions conclude that life history traits have no (Liu *et al*. 2014), or relatively minor or taxon‐specific roles (Fujisaki *et al*. 2010; van Wilgen & Richardson 2012; Allen *et al*. 2013; Tingley *et al*. 2016) as determinants of success at establishment and spread, at least in comparison to the characteristics of the introduction event, such as introduction effort, and the introduction location, such as climatic suitability (Rago *et al*. 2012; Mahoney *et al*. 2015). These studies, however, are spatially restricted, consider only few life history traits and thus ignore covariation between them that can lead to erroneous conclusions (Barton & Capellini 2011; Capellini *et al*. 2015), do not consider alternative theoretical frameworks of population growth, focus on just one or two invasion stages, or have incomplete phylogenetic control. Here we test the predictions of fast advantage and slow advantage models of how life history traits affect population growth in worldwide invasion outcomes of amphibians and reptiles from introduction to spread. We follow the same framework as our recent study of mammalian invasions (Capellini *et al*. 2015), which enables us to make a direct comparison between results across lineages and to begin drawing general conclusions on how populations grow from small sizes. We use a species level approach (Capellini *et al*. 2015) that, in comparison to population level approaches used in previous studies (see Supporting Information, section 1.1.2), is less affected by factors that influence population level outcomes, such as the degree of climate matching, which might mask the role of species’ traits. We build the largest database of herpetological life history traits to date, and combine it with a new standardised database of global herpetological invasions. Using modern phylogenetic comparative approaches, we show that in both classes, success at establishment and spread is promoted by fast life history traits, demonstrating the importance of rapid population growth.

## Methods

### Data collection

We extracted global invasion records from invasion databases (Lever 2003; DAISIE 2008; Kraus 2009; ISSG 2014), which we supplemented, cross checked and verified using a large number of additional sources (Supporting Information section 1.1.1 and 4.2). We first built an event level data set of all recorded introductions to a unique location outside the native range, at the smallest geographical scale provided in the source, for every amphibian and reptile species included in the sources. We recorded the success or failure of each alien population at the introduction, establishment and spread stages of the invasion pathway. On the basis of these data, we classified success or failure at the species level as in Capellini *et al*. (2015). Specifically, because we were interested in the intrinsic potential of a species to succeed, we considered a species successful at a stage when at least one of its alien populations met the criteria for success at that stage, scored as a binary variable (success = 1, failure = 0). A species was considered introduced if it was transported by humans and released, either intentionally or unintentionally, outside its native range into an environment where survival was not maintained by human care (i.e. excluding zoos and captive enclosures, but including human modified habitats such as urban environments). A species was judged successfully established if at least one introduced population had persisted for at least the duration of the species maximum lifespan without additional propagule pressure since the latest release, thus confirming successful reproduction and first generation survival. A species was considered successful at spread if at least one of its established populations grew substantially to support remarkable range expansion beyond the location of introduction. This was judged through descriptions of the population growth trajectory post‐establishment in multiple sources and, when available, range maps, which demonstrated an unambiguous history of spread over a large area given the introduction date. For both establishment and spread, we excluded all cases where a continuous release of individuals was likely, as with popular pet species, because this prevented judgement of whether the species had an intrinsic ability to establish a self‐sustaining population and grow remarkably post‐establishment to spread over large areas. We excluded any record that the main sources listed as introductions but which further investigation indicated were doubtful, such as records of intercepted populations that were never released, reintroductions to native range, and populations that were likely native. Two authors double checked, and if necessary discussed, the classification decisions for a random 10% of the species in the sample across all stages; all contentious records at any stage; and all cases of successful establishment. Where information was lacking, contradictory or ambiguous for all introduction locations for a given species at a given stage, we excluded the species from that and subsequent stage(s) of the analysis. Full details on the precise criteria for each stage are given in the Supporting Information section 1.1.1 and on scoring population outcomes at the species level in Supporting Information section 1.1.2.

We included in our analysis the magnitude of introduction effort (i.e. propagule pressure) for a species, given that some species are introduced more frequently than others and this factor helps to explain differences in invasion success (Lockwood *et al*. 2005). Following previous studies we quantified introduction effort at the species level as the number of unique locations each species was introduced to (Forsyth *et al*. 2004; Capellini *et al*. 2015). This measure correlated strongly and positively with the total number of individuals released across all locations in past studies (Forsyth *et al*. 2004; Capellini *et al*. 2015, Supporting Information section 1.1.3) and allowed us to analyse events where the number of released individuals was unknown, as it was the case for at least 95% of recorded herpetological introductions.

We collected life history data by combining many existing life history databases and supplementing these with additional data from the primary literature (Supporting Information section 1.1.4, references in Supporting Information section 4.3), leading to a total sample size of 5716 amphibian species and 9046 reptile species with at least one life history trait (Table S1). Due to differences in reproductive biology as well as conventions in data reporting, the traits for amphibians and reptiles partially differed. For example clutch frequency was rarely reported for amphibians so could not be included due to small sample sizes. For amphibians we included snout‐vent length (SVL, mm), egg size (ES, mm), clutch size (CS, number of eggs), age at sexual maturity (SM, years) and reproductive lifespan (RL, as the difference between maximum longevity and age at sexual maturity). For reptiles we recorded body mass (BM, g), hatchling mass (HM, g), clutch size (CS), clutches per year (CY), age at sexual maturity (SM), reproductive lifespan (RL) and parity (PA, oviparous = 0, viviparous = 1). We used female sizes for the adult body size measures (BM and SVL) when available, otherwise we used data from unspecified sex measurements. For reptiles we calculated ‘offspring value’, a measure of the value of each reproductive event relative to lifetime reproductive effort (OV = 1/(CY * RL)), with low values indicating potential for bet‐hedging (Sol *et al*. 2012, Supporting Information section 1.1.5).

### Phylogenetic analyses

**Figure 1:**
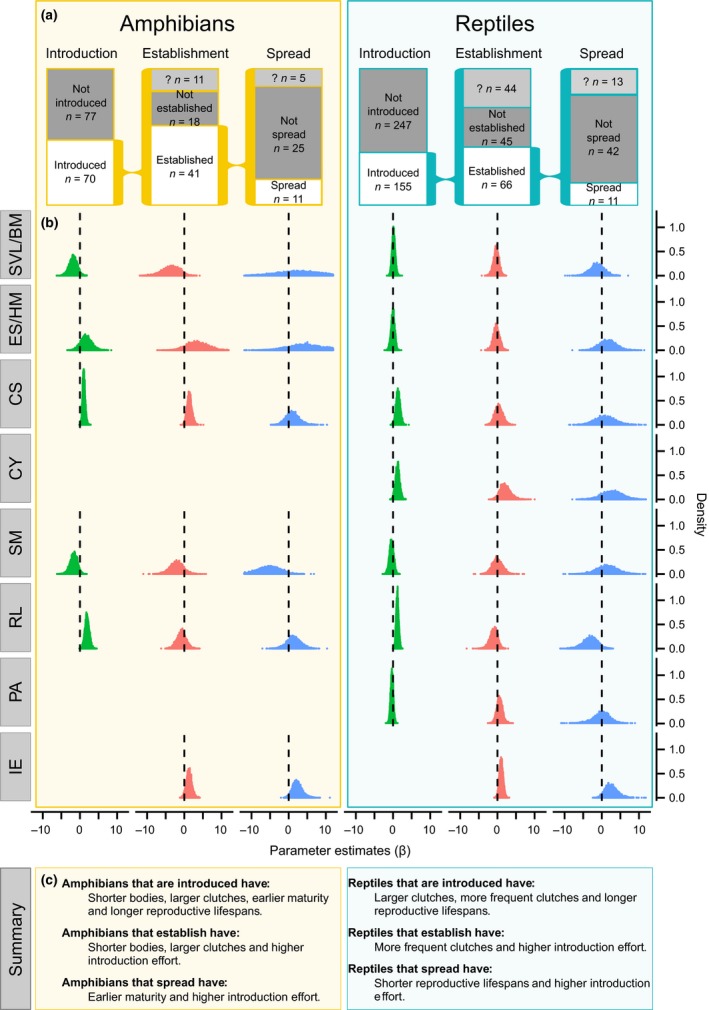
The success of amphibians and reptiles at each stage of the invasion pathway: introduction, establishment and spread. (a) The sample size and number of species that succeed or fail at each stage of invasion, including species for which no reliable judgement can be made (indicated as ‘?’). (b) Posterior distributions of the parameter estimates (β) of life history traits and introduction effort at each stage of invasion. Traits are: snout‐vent length (SVL, amphibians), body mass (BM, reptiles), egg size (ES, amphibians), hatchling mass (HM, reptiles), clutch size (CS), clutches per year (CY, reptiles only), age at sexual maturity (SM), reproductive lifespan (RL), parity (PA, oviparous = 0, viviparous = 1, reptiles only) and introduction effort (IE). Posterior distributions are centred on zero (dashed line) when they have negligible influence on the probability of success and are substantially shifted from zero when they are influential. In (c) summary of results by stage of invasion in both classes.

Our analysis benefited from recent comprehensive time calibrated molecular phylogenies of all reptile and amphibian orders (Supporting Information section 1.1.6; Jaffe *et al*. 2011; Oaks 2011; Pyron & Wiens 2011; Pyron *et al*. 2013). The ‘main’ analyses, presented here, included only the species present in these phylogenies that had complete life history data on all traits, resulting in a total sample of 147 amphibians (70 introduced) and 402 reptiles (155 introduced, Fig. 1a). The distribution of introduction effort was highly skewed in both classes, with some species being introduced many times, but most just once or twice. Log transformation did not normalise this distribution, therefore, we converted introduction effort to a binary trait by splitting it at the median number of introduction locations (> 2 or ≤ 2 locations for both classes). Results were qualitatively similar under different transformations (Supporting Information section 2.5). All other continuous traits were log_10_‐transformed, resulting in approximately normal distributions.

The binary response variable (success or failure at each stage) was modelled using phylogenetic generalised linear mixed models in a Bayesian framework (Hadfield 2010) with life history traits and introduction effort as predictors. At the stage of introduction we compared introduced species with non‐introduced species. At establishment we only analysed the subset of species that were introduced and compared those that succeeded at this stage vs. those that failed. Likewise, at spread we only analysed species that established, comparing those that remained localised with those that spread (Fig. 1a). These are the appropriate comparisons to make when testing which traits differ between unsuccessful and successful species at each stage of the invasion pathway (van Kleunen *et al*. 2010a). We used a probit model in MCMCglmm (Hadfield 2010), with largely uninformative priors (normal distribution with a mean of zero and a variance of 10^8^) for all predictors treated as fixed effects, and a chi‐squared prior for the phylogeny treated as a random effect as this best approximates a cumulative uniform distribution (Hadfield 2010; de Villemereuil *et al*. 2013). The residual variance cannot be estimated when using a binary response variable, so we followed standard procedure and fixed it to 1. We ran MCMC chains for 15 million iterations, discarded the first million as burn‐in and sampled every 2500 iterations, resulting in effective sample sizes (ESS) over 5000 for all parameters. The importance of phylogeny was assessed with heritability (*h* ^2^) (Hadfield 2010) which ranges from 0 (no phylogenetic signal) to 1 (high phylogenetic signal) and is interpreted in the same way as Pagel's λ in a phylogenetic generalised least squares framework. Trace plots of posterior distributions and ESS were examined as conventional diagnostics of chain performance to confirm that chains converged, had good mixing and acceptable, low levels of autocorrelation. All main analyses were run three times and results did not differ qualitatively between runs. Here we report the results of the first run.

The importance of each variable to success at each stage was assessed by the proportion of the posterior distribution of parameter estimates (β) crossing zero. Unimportant traits have posterior distributions of β estimates centred on zero (i.e. approximately 50% of samples cross zero), whereas important traits have posterior distributions clearly shifted away from zero and not substantially overlapping zero. To translate the magnitude of effects from the β estimates we calculated average partial effects (APEs; Long 1997; Greene 2012). APEs estimate the probability of change between success and failure at an invasion stage for a unit change in a given independent variable, averaged across all observed values of all other independent variables in the model. We calculated APEs at each iteration of the posterior β distribution and took the mean APE from the resulting APE posterior distribution and converted this from the log scale back to the original units.

We checked the robustness of our results with several additional analyses. Conclusions were not affected by potential multicolinearity between predictors. In amphibians all variance inflation factors (VIFs, Supporting Information section 1.2.2) were below the critical threshold of five. In reptiles, covariance between life history traits led to body size having a VIF score over five. Removing body size from the full model and rerunning the analysis without this variable (‘reduced models’) produced qualitatively similar results to those presented here (Supporting Information section 2.1). We also checked whether our sample of species with complete life history data was a random sample with respect to the life history traits of all amphibian or reptile species included in the phylogeny, by comparing the trait values of species with complete life history data to species with partial life history data using phylogenetic *t*‐tests (Supporting Information section 1.2.4). We found minor sampling effects between species with full and partial life history data (Supporting Information section 2.3), however, these did not affect our conclusions (Supporting Information section 2.4). Specifically, our results did not qualitatively differ at the stages of establishment and spread when we ran additional analyses for single life history traits as predictors of invasion outcomes using all species (i.e. with full or incomplete life history data), indicating that our conclusions were robust.

## Results

We first investigate whether alien amphibian and reptilian species differ in life history traits from species that have never been introduced. If present, differences indicate biases in likelihood of introduction in favour of species with particular traits. Introduction biases can therefore influence the sample of species included in analyses of subsequent invasion stages (van Kleunen *et al*. 2010a; Capellini *et al*. 2015). We find strong introduction biases in both taxonomic classes. Among amphibians (*n *=* *147 species), the 70 introduced species have smaller snout‐vent lengths (3.7% of the β posterior distribution crossing zero), larger clutches (< 0.1%), earlier maturity (3.9%) and longer reproductive lifespans (< 0.1%) than the 77 non‐introduced species (Fig. 1b). APEs indicate that, on average, the probability of being introduced is 3.5% higher per mm decrease in snout‐vent length, 2.1% greater for every additional egg laid, 3.2% increased for bringing sexual maturity forward a year, and 3.8% greater for each additional year of reproduction. In reptiles (*n *=* *402 species) the 155 introduced species have larger clutches (1.1% β crossing zero), more frequent breeding (1.6%) and longer reproductive lifespans (< 0.1%) than the 247 non‐introduced species. Using APEs, we calculate that this corresponds to increases of 2.0, 1.8 and 1.8% in the probability of introduction for each additional egg per clutch, clutch per year and year of reproductive lifespan respectively.

From the pool of introduced species, we test the key predictions made by alternative models of how life history traits support population growth at the establishment stage in amphibians (*n* = 59; 41 established, 18 not established) and reptiles (*n *=* *111; 66 established, 45 not established). Smaller bodied amphibians (4.1% β crossing zero) and those with larger clutch sizes (1.4%) are more likely to establish. APEs show that, on average, a one mm decrease in body length increases the probability of establishment by 6%, and a one egg increase in clutch size makes establishment 2.3% more likely. In reptiles successful establishment is associated with more frequent clutches (4.5% posterior crossing zero), so that each additional clutch per year increases the probability of establishment by 2.8% as estimated with APEs. As expected, in both amphibians and reptiles, species are more likely to establish when they have been introduced to more locations (amphibians: 3.8% crossing zero, reptiles: 2.0%).

Fast life history traits also promote success at the stage of spread. In amphibians (*n *=* *36; 11 spread, 25 not spread), early maturity (2.8% posterior crossing zero) associates with success; APEs estimate that achieving maturity a year earlier increases the probability of spread by 4.4% on average. In reptiles (*n *=* *53; 11 spread, 42 not spread) spread is associated with short reproductive lifespans (4.6% β crossing zero). Every additional year of reproduction decreases the probability of spread by 1.6%. The importance of increased introduction effort on success remains at the spread stage in both classes (amphibians: 2.6% β crossing zero, reptiles: 4.1%).

In reptiles, offspring value, a measure of the relative importance of each clutch to lifespan reproductive success, is lower in introduced species (< 0.1% crossing zero). However, alien reptiles with low offspring values are not more likely to establish (33.3% β crossing zero) or spread (29.0% β crossing zero). This introduction bias is an outcome of how offspring value is calculated from clutch frequency and reproductive lifespan (Supporting Information section 2.2, Table S5), as we also find that species with more frequent clutches and longer reproductive lifespans are more likely to be introduced. We cannot investigate the role of offspring value in amphibians because of a lack of data on clutch frequency (see Methods, Data collection).

**Figure 2:**
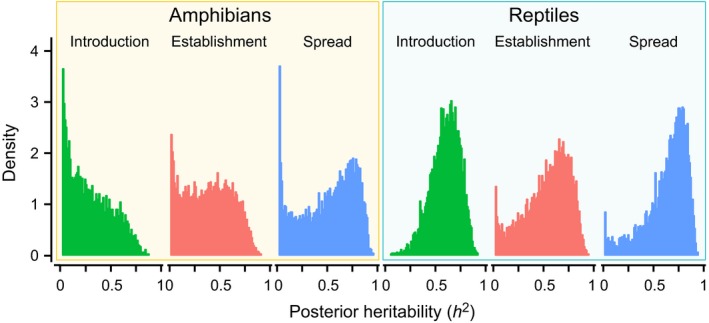
Posterior distribution of heritability (*h* ^2^) at each stage of the invasion pathway in amphibians and reptiles.

Heritability scores are relatively high at all stages of the invasion pathway in reptiles (introduction mean *h* ^2^ = 0.57, establishment = 0.56, spread = 0.67; Fig. 2), indicating an important role for shared ancestry in the relationship between life history traits and biological invasions. Heritability scores in amphibians are slightly lower, but still demonstrate the necessity of appropriately accounting for phylogeny, (introduction mean *h* ^2^ = 0.20, establishment = 0.37, spread = 0.54; Fig. 2).

## Discussion

Competing theoretical models differ in whether they predict that species with fast or slow life history traits are more successful invaders. Fast life history traits should support invasion by allowing small populations to grow quickly, thus reducing the period during which they are vulnerable to demographic and environmental stochasticity (Pimm 1991). In contrast, recent models predict that ‘slow’ species are better able to avoid extinction because they do not experience drastic declines in population size in response to negative demographic (Jeppsson & Forslund 2012; Sæther *et al*. 2013) and environmental stochastic events (Sæther *et al*. 2013). Our results show that there is a clear advantage for traits associated with a fast life history strategy in alien herpetofauna (Fig. 1c). Small body size and large clutches promote establishment success in amphibians, and frequent clutches promote establishment success in reptiles. Early maturity in amphibians and short reproductive lifespans in reptiles promote success at spread. While the specific traits differ between classes and stages of invasion, they support predictions of classic fast advantage models of small population growth (Pimm 1991). These results are also in agreement with empirical findings in mammals (Capellini *et al*. 2015) and plants (Richardson & Rejmánek 2004; van Kleunen *et al*. 2010b), and some previous studies of amphibian and reptile invasions (Fujisaki *et al*. 2010; van Wilgen & Richardson 2012; Mahoney *et al*. 2015). We find that the magnitude of introduction effort is a strong predictor of success at the global scale at both establishment and spread in agreement with previous studies (Forsyth *et al*. 2004; Rago *et al*. 2012; Sol *et al*. 2012; van Wilgen & Richardson 2012; Capellini *et al*. 2015; González‐Suárez *et al*. 2015; Mahoney *et al*. 2015), but our results also reveal an important role of life history traits in explaining species’ differences in invasion outcome.

Our findings contrast with evidence in birds, where successful establishment is weakly associated with small clutches and strongly associated with low brood (offspring) value (Sol *et al*. 2012), traits generally linked with slow strategies that prioritise future over current reproduction, and consistent with slow advantage models of population growth. Low offspring value indicates that reproductive effort is divided into many attempts and suggests that a bet‐hedging strategy, that reduces the fitness consequences of bouts of reproductive failure, is particularly important in avian invasions (Sol *et al*. 2012). In reptiles (this study) and mammals (Capellini *et al*. 2015), however, offspring value is not associated with success at establishment or spread. Here we discuss how these seemingly contrasting results in birds and non‐avian tetrapods could be reconciled.

The exceptionally long reproductive lifespans of birds relative to their body size (Healy *et al*. 2014) may explain why they are the only class where no strong relationship between increased fecundity and invasion success is observed (Sol *et al*. 2012). Jeppsson & Forslund's (2012) slow advantage model shows that different combinations of fast and slow life history traits interact to determine a population's response to demographic stochasticity. Specifically, increased fecundity is associated with higher extinction risk for long‐lived but not short‐lived species, suggesting that high fecundity should decrease invasion success only in long‐lived species. In addition, long reproductive lifespans are associated with a suite of traits that decrease adult mortality, directly or indirectly, such as flight (Healy *et al*. 2014), large brain size (Barton & Capellini 2011) and behavioural plasticity (González‐Lagos *et al*. 2010). Birds may therefore have a greater capacity to explore the environment and develop behavioural responses to novel challenges (Sol & Maspons 2016), and thus be the only vertebrate lineage in which slow life history traits are advantageous (Sol *et al*. 2012; Sol & Maspons 2016). Ectothermy in reptiles and amphibians is considered an alternative way of achieving flexibility as ectothermy frees organisms from constraints, such as the need to acquire food to produce heat, and allows the adoption of opportunistic life history strategies that adjust to prevailing environmental conditions (Shine 2005). If ectothermy affords flexibility that leads to similar benefits to those expected in birds, we should find that the association between invasion success and life history traits is more bird‐like than mammal‐like in amphibians and reptiles. However, we find no support for this suggestion.

A recent model of population growth in plant invasions that incorporates transient dynamics (Iles *et al*. 2016) may also help reconcile the contrasting results between birds and the other terrestrial vertebrate classes. Transient dynamics describe how a population growth rate changes over the short term in populations with unstable stage (or age) structures as they transition towards long term (asymptotic) stable population dynamics. Transient dynamics are important in invasions because introduced populations are generally small, have an unstable stage structure, and stochastic events do not affect all stage classes equally (Iles *et al*. 2016). Importantly, transient dynamics are highly dependent on life history traits (Gamelon *et al*. 2014; Iles *et al*. 2016). For a given starting population size, species with fast life history traits generally have lower transient population growth rates relative to their asymptotic growth rates compared to slow species, especially when the founder population is primarily composed of individuals at early life stages (Iles *et al*. 2016). Therefore, founder populations of fast species primarily composed of individuals at early life stages suffer high risk of extinction. However, when populations of fast species are biased towards later life stages they grow rapidly because they produce many offspring that mature quickly, thereby greatly improving the short‐term population growth rate relative to the long‐term growth rate (Koons *et al*. 2006). Iles *et al*.'s (2016) model therefore highlights the importance of considering the stage structure of introduced alien populations when investigating success at establishment and spread. While plant invasions often initiate as seeds (Richardson & Rejmánek 2004), terrestrial vertebrates are almost always introduced at later life stages – we did not record any cases of amphibians or reptiles that established from introductions of eggs, for example (sources in Supporting Information section 4.2). Thus, fast traits should be of particular benefit to short‐lived species introduced as adults, but less so to relatively long‐lived species (such as birds) when they are introduced as adults (Iles *et al*. 2016), potentially explaining why fast traits are advantageous in mammals, reptiles and amphibians, but not birds.

Mortality of juveniles relative to adult mortality is an additional key demographic factor that can potentially explain differences in invasion success among taxa. Specifically, in amphibians, reptiles and short‐lived mammals (e.g. rodents), juvenile mortality relative to adult mortality is high compared to birds and long‐lived mammals (Jones *et al*. 2013). As a result, natural populations of reptiles, amphibians and short‐lived mammals are generally heavily skewed towards early life stages and away from adults compared to birds and long‐lived mammals (Jones *et al*. 2013). This concentrates reproductive value in proportionally fewer adults that contribute relatively more than younger individuals to population growth following a negative environmental event (Koons *et al*. 2006). Thus, reptiles, amphibians and short‐lived mammals should benefit more from fast traits and less from the dampening effect of slow life history traits and strategies such as bet‐hedging, than birds and long‐lived mammals.

Finally, we show that introduction biases are highly consistent between reptiles, amphibians, mammals (Capellini *et al*. 2015) and birds (Jeschke & Strayer 2006): preferences for introducing species with longer reproductive lifespans and larger clutches/litters are observed in all taxa; frequent breeding events in reptiles, mammals and birds; and early maturity and small body size in amphibians. The differences in amphibians may result from the absence of data on clutch frequency in this group and patterns of covariation between life history traits. These biases, a mix of fast and slow traits, may be the consequence of human preferences for introducing animals that are ‘hyper‐productive’, having high fecundity over long lifetimes, traits that can increase economic value (Pyšek & Richardson 2010). Although we do not distinguish between accidental and intentional introductions in this study, approximately half of amphibian and two thirds of reptile introductions we record are accidental, suggesting that these biases are likely to operate via both pathways. Overall, these biases match the associations at establishment and spread, strengthening the conclusion that in general humans preferentially introduce species with life history characteristics that subsequently facilitate establishment and spread of alien populations (Capellini *et al*. 2015). The biases observed in reptiles, amphibians and mammals (Capellini *et al*. 2015) also raise the possibility that ‘slow’ species in these lineages, which are filtered out at the stage of introduction, might show more bird‐like characteristics of success at the stages of establishment and spread.

To conclude, our results show that both reptiles and amphibians have key life history traits associated with success at each stage of biological invasions. Results at the stages of establishment and spread support an emerging consensus that species do differ in their intrinsic potential to grow from small populations and invade new locations (Sol *et al*. 2012; Capellini *et al*. 2015), counteracting a trend in recent years to downplay the importance of species level demographic traits in biological invasions, both in general (Hayes & Barry 2008), and in reptiles and amphibians in particular (Mahoney *et al*. 2015). We find that the way life history traits support biological invasions is broadly consistent across amphibians, reptiles and mammals, showing that fast lived species are more likely to establish and spread than slow lived ones. Birds, however, exhibit different life history strategies that support population growth in invasions, being most successful when they invest relatively little in each reproductive event (Sol *et al*. 2012). While differences between classes may be explained by differences in lifespan, brain size and behavioural flexibility (González‐Lagos *et al*. 2010; Healy *et al*. 2014; Sol & Maspons 2016), a recent model incorporating transient dynamics in how species with different life history strategies grow from small numbers during invasions (Iles *et al*. 2016), offers an additional framework that can potentially unify these results. Our study emphasises the need to pay particular attention to mammals, reptiles and amphibians with fast life history traits that are released or begin to establish, as the increased likelihood that these populations will expand rapidly, and potentially lead to large scale and severe environmental impacts, implies that early intervention is likely to be an especially efficient use of resources (Pyšek & Richardson 2010).

## Author contributions

I.C. conceived and led the research, W.L.A., I.C. and S.E.S. designed the study, W.L.A. and S.E.S. collected the data, W.L.A. analysed the data and drafted the manuscript, all authors contributed substantially to revising and preparing the manuscript; I.C. advised on and supervised all stages of the research.

## Data Availability

All data supporting the results is available from the Dryad Digital Repository: http://dx.doi.org/10.5061/dryad.2d7b0
