# Supplementary material for: Fast life history traits promote invasion success in amphibians and reptiles
Source: Ecol Lett. 2017 Jan 4;20(2):222–30. doi: 10.1111/ele.12728 (PMC6849728; doi:10.1111/ele.12728)
Supplement: Supplementary file 1 [file ELE-20-222-s001.docx]

# Fast life history traits promote invasion success in amphibians and reptiles – supplementary information

1. **SUPPLEMENTARY METHODS 2**
   1. **Data collection** 2
      1. Classification of the status of alien species 2
      2. Classification of invasion success at the species level 6
      3. Introduction effort (propagule pressure) at the species level 8
      4. Life history data 10
      5. Offspring value 11
      6. Amphibian and reptile phylogenies 12
   2. **Statistical analyses** 12
      1. Average partial effects 12
      2. Robustness of the results to multicollinearity 13
      3. Relationship between offspring value and other life history traits 14
      4. Potential sampling effects 14
2. **SUPPLEMENTARY RESULTS 15**
   1. **Multicollinearity: variance inflation factors (VIF) and reduced models** 15
   2. **Offspring value and life history traits** 18
   3. **Potential sampling effects** 19
   4. **Individual predictor models of invasion success** 21
   5. **Alternative transformations of introduction effort** 22
3. **SUPPLEMENTARY DISCUSSION 28**
4. **SUPPLEMENTARY REFERENCES 29**
   1. **Supplementary information references** 29
   2. **Data references: Biological invasions database** 32
   3. **Data references: Life history databases** 34
5. **SUPPLEMENTARY METHODS**
   1. **Data collection**
      1. **Classification of the status of alien species**

For each invasion stage - introduction, establishment and spread (Blackburn *et al.* 2011) - we classified a species as successful if at least one population of the species had been successful at that stage, following the protocol developed in Capellini *et al.* (2015). The stage of capture and transport was not considered in this study as this affects many more species, the large majority of which have not been introduced in the wild outside their native range and never go on to become alien species (e.g. zoo animals). Thus barriers at the stage of transport are separate from our question of what affects the intrinsic potential of a species to grow from a small population and become invasive.

Introduction

A population was classified as successfully introduced only if it was released by humans into the wild, either intentionally or accidentally, outside of the species’ native range (Blackburn *et al.* 2011). For the purpose of this study, we did not distinguish between accidentally and intentionally introduced species. The spatial resolution of the introduction location was recorded as the smallest unit described in the source, whether a precise location, city, province or country. Species that were described as ‘exotic’, ‘alien’, ‘invasive’, ‘introduced’, ‘naturalized’, ‘released’, ‘non-native’ or similar were classified as introduced only if they met this definition. Introductions to areas managed by humans were only considered when the animals were free ranging and not provisioned. We discounted cases where it could not be determined that the potential introduction was outside the native range from descriptions and range maps. This included reintroductions to areas where the species was formerly present (i.e. historical native range) and introductions to islands within the native range. Unconfirmed introductions were also discounted, as were species that were described as alien but potentially arrived without direct human assistance (e.g. natural dispersal or range changes due to climate-change). A species was classified as successful if there was unambiguous evidence of at least one alien population as defined above.

In total we identified 138 introduced amphibian species and 352 introduced reptile species, though complete life history records were not available for all these taxa (see section 1.1.4). Specifically, we had complete life history data for a total of 147 amphibian and 402 reptile species, including both introduced and not introduced species, and of these 70 amphibians and 155 reptiles were classified as successfully introduced.

Establishment

We classified all introduced reptile and amphibian populations for successful establishment, defined as a self-sustaining population (Blackburn *et al.* 2011) which had persisted for at least the maximum lifespan of the species. This criterion confirmed successful reproduction of the founder population and first generation survival. We thus checked the elapsed time between the date of introduction to a locality, the last known date of presence, and the maximum lifespan recorded in the life history database. When a range was given for introduction dates we remained conservative and used the latest date. When there was no date of introduction we used the date of the first reference to the introduction in the literature. If there had been multiple introductions to the same locality we used the date of the last introduction. Remaining cases for which this lower bound on introduction date could not be set were discarded from the establishment and spread stages. Introductions that were known to have begun hundreds or thousands of years ago, but which lacked an exact date, were recorded as successes if the information was unambiguous and the population persisted. Subsequent eradication by human efforts after introduction was not counted as failure to establish if the population survived for at least the time period equivalent to the species’ maximum lifespan prior to eradication; in this instance the case was counted as a success. Introductions with unknown or unclear outcomes were discarded, for example when repeat releases prevented determination of whether the population was self-sustaining. We classified a species as successful at establishment if at least one of its alien populations had successfully established as defined here.

Of the 138 introduced amphibians, we recorded 74 species in total that successfully established and 25 that failed to establish, of which 41 and 18 species respectively had complete life history records and were included in the main analysis of establishment success (Fig. 1a, main text). Of the 352 introduced reptiles we recorded 123 species as successfully established in at least one location and 91 species as having failed to establish a self-sustaining population, of which 66 successful and 45 unsuccessful species had complete life history information (Fig. 1a, main text). The remaining 39 introduced amphibians and 138 introduced reptiles had insufficient information to be confidently classified at this stage of invasion.

Spread

At the stage of spread we judged each of the successfully established populations as having successfully spread if it exhibited remarkable range expansion from the initial location of introduction (van Kleunen *et al.* 2010a, b; Blackburn *et al.* 2011). In descriptive accounts of introduction events in the sources, suggestive language such as ‘widespread’, ‘common’, ‘expanding’, ‘flourishing’, ‘pest’, ‘is now common everywhere’ and similar, was taken as starting evidence of spread, which we followed up with targeted searches on the outcome of the introduction in other sources. To confirm spread as successful we required more detailed information on the extent of the range expansion and/or detailed description of how the population had expanded. When recent range maps of the extent of invaded range were available we used these to update original records on whether range expansion had been remarkable given the date of introduction. We discarded cases of potential spread on the basis of apparent wide distribution alone if there was indication that such apparent spread could have been achieved by multiple releases over a wide area; we considered evidence of successful spread only cases where a single introduced population to a unique location had shown unambiguous remarkable expansion. Populations described as ‘restricted’, ‘not expanding’, ‘localized’, ‘small’ and similar were judged as unsuccessful at this stage. Cases of range expansion across small islands (<50,000km^2^) were discarded as we considered the potential area too small to unambiguously demonstrate that a species had intrinsic potential to invade. However if a population had a limited range on a small island, as described by wording indicated above, this was counted as a failure to spread. We considered successful at spread three populations that achieved extraordinarily high population densities on islands (*Anolis carolinensis* on the Ogasawara Islands, *Boiga irregularis* on Guam, and *Eleutherodactylus coqui* on Hawaii Island). Ambiguous cases were thoroughly investigated by two authors, considered independently, then discussed by all three authors, and included if only consensus was attained, otherwise they were discarded from the analysis of outcomes at the stage of spread. After classifying spread for each introduced population, we classified spread at the species level and judged a species successful when at least one established population met the criteria for success at this stage.

Of the 74 successfully established amphibian species and 123 successfully established reptile species, 15 amphibian and 12 reptile species successfully spread and 43 amphibian and 70 reptile species failed to spread. Of these, 11 successful and 25 unsuccessful amphibians, and 11 successful and 42 unsuccessful reptiles, had complete life history data. Information was not sufficient to classify 16 established amphibians and 41 established reptiles for success at the stage of spread.

Sources and taxonomy

We used four main sources to initially construct the database of reptile and amphibian introductions at the location level (Lever 2003; DAISIE 2008; Kraus 2009; Invasive Species Specialist Group ISSG 2014). We then supplemented, verified and further investigated records using additional sources (full data reference list in section 4.2.). Data were initially compiled by two authors. To ensure consistency each author cross-checked a random 10% of all records and all records that had ambiguous outcomes under our protocol. Further checks were made during classification at the species level. When necessary, species names were updated to the taxonomies of The Reptile Database (Uetz & Hošek 2015) and Amphibian Species of the World (Frost 2015).

- - 1. **Classification of invasion success at the species level**

The purpose of the present study is to understand the intrinsic potential of amphibian and reptile species to succeed in novel environments given their life history traits, rather than what characteristics of a particular introduction event lead to success or failure, or the relative importance of event, location and species level characteristics (Mahoney *et al.* 2015). We thus followed the approach of Capellini *et al.* (2015) and analysed overall potential to succeed or fail at the species level as described above (see section 1.1.1).

The species level approach is justified by previous findings in a wide variety of taxa, including amphibians and reptiles, that (i) species identity is one of the strongest predictors of invasion success in taxonomically based studies across species that include multiple introductions for a species; (ii) a species’ history of successful invasion elsewhere in the world predicts success for that species in a specific region; and (iii) failures in one location of otherwise successful alien species elsewhere are more often due to unsuitable habitats (Duncan *et al.* 2001; Forsyth & Duncan 2001; Forsyth *et al.* 2004; Marchetti *et al.* 2004; Bomford *et al.* 2009; Tingley *et al.* 2011; Rago *et al.* 2012; Richardson & Pyšek 2012; Sol *et al.* 2012). This evidence demonstrates that species vary in their intrinsic potential to grow from small numbers and become widespread, supporting the validity of using a species level approach to understand the role of species’ traits, as we do here to identify which life history traits explain differences across species in relation to invasion outcomes. Conversely, a population level analysis of this question would be affected by peculiarities of the introduction events and introduction locations, such as the degree of climate matching, that might mask important species level effects (Capellini *et al.* 2015). A species level approach can therefore better reveal a species’ potential to invade, regardless of whether or not some of its populations fail because of introduction into less suitable environments (Forsyth *et al.* 2004). An important advantage of our approach is that classifying success at the species level allows incorporation of appropriate phylogenetic controls.

In phylogenetic comparative studies the unit of analysis is the species. Including population level introduction outcomes in a comparative analysis across species involves modelling within species variation as an additional random effect. Simulations show that including within species variation in comparative analyses across species only slightly improves parameter estimates, but does not change the direction or importance of fixed effects (Ives *et al.* 2007; Revell & Reynolds 2012). However, including population level invasion outcomes as within species variation in our study would dramatically reduce sample sizes because at the population level introduction effort cannot be measured as number of introduction locations (see section 1.1.3), since each location would correspond to an alien population. In other words, this would make location identity and population identity identical and consequently unsuitable for use as both a dependent variable (population outcome) and a predictor (introduction effort). As a result, a different measure of introduction effort would be required at the event level, such as the number of introduced individuals or the number of introduction attempts (see section 1.1.3). However, quantitative information on these measurements is rarely available for amphibian and reptile introductions using either of them would therefore drastically reduce the number of species for which introduction effort could be measured (by approximately 75% at each invasion stage; see section 1.1.3). The consequence would ultimately be a substantial loss of power, as species is still the effective unit of analysis in a comparative study; within species variation being analogous to repeated measures. The small sample size of species would prevent us from testing the predictions of two alternative theoretical models of population growth that require inclusion of multiple life history traits in a single model to account for the correlated evolution between life history traits, whilst also controlling for introduction effort and estimating the effect of phylogeny simultaneously. Since the number of introduced individuals is only available for multiple populations of 7 amphibian and 6 reptile species, there would also be little advantage to including within species variation.

Finally, recent studies show that models based on native climatic niche data may fail to predict whether an alien population can establish and spread given the suitability of the novel range relative to the native range, and that remarkable ‘niche shifts’ may occur, including several examples in reptiles and amphibians (e.g. Broennimann *et al.* 2012; Di Febbraro *et al.* 2013; Li *et al.* 2014; Tingley *et al.* 2016). These recent studies show that the relative importance of climate matching in invasion success is lower than previously assumed, providing further evidence in support of a species level approach.

- - 1. **Introduction effort (propagule pressure) at the species level**

A consistent and expected finding of studies of invasion outcomes is that greater introduction effort (also termed ‘propagule pressure’) increases the chances of successfully establishing (Lockwood *et al.* 2005; Colautti *et al.* 2006; Jeschke & Strayer 2008; Richardson & Pyšek 2008; Simberloff 2009; Sol *et al.* 2012; Duncan *et al.* 2014; Capellini *et al.* 2015). The magnitude of introduction effort must therefore be controlled for in any analysis of intrinsic invasion potential; some species are introduced with much greater intensity than others and this is expected to help them succeed. Failure to incorporate introduction effort in statistical models can lead to incorrect estimates of the effects of other predictors on success at establishment and spread (Cassey *et al.* 2004).

One common measure of introduction effort is the total number of individuals released across all introductions events to a single location (Lockwood *et al.* 2005). However, for approximately 95% of recorded introduction events in amphibians and reptiles, the number of individuals released is unknown. In past studies on invasion success in amphibians and reptiles, this problem has been addressed by using proxies for the total number of individuals such as counting the number of introduction events per region (Bomford *et al.* 2009; Kraus 2009; Tingley *et al.* 2011; van Wilgen & Richardson 2012; García-Díaz *et al.* 2015), measuring import quantity (Fujisaki *et al.* 2010; García-Díaz *et al.* 2015), using the intentionality of the introduction (Rago *et al.* 2012), measures which are also often unknown, or by restricting the dataset to introductions that only occurred once (Liu *et al.* 2014). Here we followed an alternative approach that maximised the sample size of species for which introduction effort could be quantified by estimating the number of unique introduction locations a species had been introduced to (Forsyth & Duncan 2001; Forsyth *et al.* 2004; Křivánek *et al.* 2006; Capellini *et al.* 2015).

Crucially, the number of unique introduction locations is positively and strongly correlated with the total number of individuals released across all locations (Duncan *et al.* 2001; Cassey *et al.* 2004; Forsyth *et al.* 2004; Capellini *et al.* 2015), supporting the validity of this measure. This, together with evidence showing that the number of introduction locations strongly correlates with invasion success (Duncan *et al.* 2001; Cassey *et al.* 2004; Forsyth *et al.* 2004; Capellini *et al.* 2015) demonstrates that it is a good measure of introduction effort in comparative studies at the species level. Recording introduction effort as the total number of unique locations a species has been introduced to worldwide also maximises our sample size by allowing all location level data to be used (Capellini *et al.* 2015; SI 1.1.2.).

- - 1. **Life history data**

We combined records from 41 existing reptile and amphibian life history databases and supplemented these with additional records from single species studies or smaller comparative datasets (references SI 4.3.). After importing data we identified equivalent traits across datasets and standardised the units of measurement as necessary. We updated species names in all datasets to those used in the taxonomies of 12^th^ August 2015 in The Reptile Database (Uetz & Hošek 2015) for reptiles, and 13^th^ September 2015 in Amphibian Species of the World 6.0 (Frost 2015) for amphibians. We disregarded any records that were not actual biological measurements but estimates of trait values, e.g. from allometric analyses. We carefully filtered out errors by (i) checking all outliers for accuracy in histogram plots, (ii) plotting traits against body size measures and checking outliers for accuracy; and (iii) plotting traits on phylogenies and checking unusual points. Some outliers were caused by errors in source databases that were apparently the result of mistakenly recording points in the wrong unit (e.g. a few species recorded in kg when other species were recorded in grams), which we corrected by investigating records in primary sources when possible, otherwise they were removed. Apparent outliers were retained in the database after their accuracy was confirmed by follow-up checks in primary sources. Any outliers that we were not able to verify or resolve were removed from the database.

In total we collected 110,746 records. When we had multiple records of the same trait for a species, we calculated a species’ trait average by taking the mean of unique records. The number of species trait means are given in table S1.

**Table S1** The total number of species for each trait in the life history database after averaging across repeat measurements. ‘Total N species’ includes species that are not present in the phylogenetic trees and ‘Species in phylogeny’ is the sample size of species that are present in the phylogeny (SI 1.1.6). The traits are as follows: snout-vent length (SVL), body mass (BM), egg size (ES), hatchling mass (HM), clutch size (CS), clutches per year (CY), sexual maturity (SM), longevity (LG) and parity mode (PA). Scarcity of CY data for amphibians meant it was not analysed. Age at sexual maturity and longevity were used to calculate the reproductive lifespan of the species which was used in analyses. The number of species that are present in the phylogeny and have complete life history data on all analysed traits, is 147 amphibians and 402 reptiles.

|  | **Amphibians** | | **Reptiles** | |
| --- | --- | --- | --- | --- |
| **Trait** | **Total N species** | **Species in phylogeny** | **Total N species** | **Species in phylogeny** |
| SVL/BM | 2085 | 1462 | 3797 | 2588 |
| ES/HM | 963 | 838 | 2347 | 1712 |
| CS | 1018 | 875 | 3352 | 2384 |
| CY | 99 | 79 | 1424 | 1122 |
| SM | 240 | 234 | 785 | 653 |
| LG | 278 | 262 | 1656 | 1281 |
| PA | - | - | 8549 | 4165 |

- - 1. **Offspring value**

Offspring value (OV; Bókony *et al.* 2009) estimates the degree to which investment in current reproduction is prioritised over future reproduction, calculated as:

$$\mathbf{Equation 1:} OV = \frac{1}{(Clutches per year)*(Reproductive lifespan)}$$

Where a high offspring value means that reproductive effort is concentrated into a few attempts, whereas a low offspring value means reproduction is spread across many events. Low offspring value can be achieved by both short lived species reproducing very frequently and long lived species that reproduce regularly throughout their lifespans. In birds offspring value is both an independent axis of life history variation and the component of a species’ life history strategy that most strongly relates to establishment success, with species exhibiting low offspring values being more likely to establish (Sol *et al.* 2012). In mammals, however, offspring value does not represent an independent axis of life history variation, nor is it related to invasion outcomes (Capellini *et al.* 2015).

Here we examined whether offspring value had a role in invasion success in reptiles (main text) and how it associated with life history traits (sections SI 2.2). In amphibians clutch frequency was rarely recorded so we were unable to calculate offspring value.

- - 1. **Amphibian and reptile phylogenies**

For the amphibian analyses we used the time-calibrated phylogeny of Pyron & Wiens (2011). As there was no single published tree that encompasses all four extant reptile orders, we joined three separate trees of Rhynchocephalia and Squamata (Pyron *et al.* 2013), Crocodilia (Oaks 2011) and Testudines (Jaffe *et al.* 2011) using the topology and node depths in the TimeTree of Life (Shedlock & Edwards 2009). This placed the split between Testudines and Crocodilia at 230.7 Ma, and the split between this clade (Archosauromorpha) and the Rhynchocephalia/Squamata clade (Lepidosauromorpha) at 274.9 Ma.

- 1. **Statistical analyses**
     1. **Average partial effects**

We used average partial effects (APEs; Long 1997; Greene 2012) to measure the effect size of life history traits on outcomes at each invasion stage. APEs are estimated from the posterior distribution of *β* values using the following equation (Greene 2012):

$$\mathbf{Equation 2}: APE of x_{c}= \sum_{i=1}^{n} \frac{f(x_{i}^{'}\hat{\beta})\hat{\beta_{c}}}{n}$$

Where $f$ is the normal density function (*ϕ*) for a probit model, $\hat{\beta_{c}}$ is the parameter estimate of a given life history variable $x_{c}$, ($x_{i}^{'}\hat{\beta})$ are the fitted model values for each independent variable, and $n$ is the number of observations. Equation 2 estimates the contribution of the parameter estimate ($\hat{\beta_{c}}$) for a life history variable ($x_{c}$) to the fitted model values ($x_{i}^{'}\hat{\beta})$at each observed value of all other independent variables in the model, thus it quantifies the average partial effect of $x_{c}$ at that iteration (Mood 2010). In a Bayesian framework, APEs are calculated at each iteration of the posterior distribution of *β* estimates, resulting in a posterior distribution of APEs for each independent variable.

- - 1. **Robustness of the results to multicollinearity**

Life history traits covary with each other in amphibians and reptiles (Dunham & Miles 1985; Bauwens & Diaz-Uriarte 1997; Clobert *et al.* 1998). Multicollinearity between predictors therefore represents a potential problem in multi-predictor models that we assessed using variance inflation factors (VIF). VIFs quantify the amount of variance that is increased due to the presence of multicollinearity (Quinn & Keough 2007).

VIFs were calculated for all life history traits and introduction effort using non-phylogenetic GLS regressions as:

$${\mathbf{Equation 3:} VIF}_{k}=\frac{1}{(1- {R_{k}}^{2})}$$

Where R_k_^2^ is the R^2^ value obtained by regressing the *k^th^* predictor on the remaining predictors. VIF values greater than 5 are considered evidence of possibly problematic collinearity and greater than 10 of strong collinearity (Quinn & Keough 2007). Using non-phylogenetic analyses is a conservative approach because the strengths of associations between life history traits are reduced when phylogeny is taken into account, which would reduce VIFs.

If possibly problematic VIFs greater than 5 were found, we removed the variable with the highest VIF from the model and recalculated VIFs for the reduced model. If any VIFs over 5 still remained, we removed the variable with the highest VIF score in this model, and repeated the procedure until a ‘reduced model’ that contained all the predictors with VIFs lower than 5 remained. We next repeated the main MCMCglmm analyses modelling success at each stage of invasion using these ‘reduced models’. These models are reported in section 2.1.

- - 1. **Relationship between offspring value and other life history traits**

In reptiles we assessed whether OV (see 1.1.5) represented a relatively independent axis of life history evolution by testing the relationship between OV and each life history trait using phylogenetic generalised linear models in MCMCglmm (Hadfield & Nakagawa 2010). The life history traits were treated as fixed effects with normally distributed priors with a mean of 0 and a large variance around the mean (10^8^) (Hadfield & Nakagawa 2010; Hadfield 2012). The phylogeny was treated as a random effect with a proper Cauchy prior with wide scaling variance (10^8^) (Hadfield & Nakagawa 2010; Hadfield 2012). The MCMC chains were run as described in the main text with each stage of invasion run separately. The results are reported as the mean and SD of the posterior distribution of *β* estimates for the slope of the pairwise association between OV and each life history traits, and the percentage of the *β* posterior distribution crossing over 0.

- - 1. **Potential sampling effects**

Species that did not have complete life history data were not included in the main analyses. We therefore checked whether the sample of species for the main analysis represented a random sample of all reptile or all amphibian species. We tested whether the mean life history traits differed between the species with all life history data and included in the main analysis, and those of species with partial life history data not included in the main analysis. To this end we used phylogenetic t-tests (Organ *et al.* 2007) in the R package *caper* package (Orme 2013) with the life history trait of interest as the dependent variable and the classification of each species as ‘included’ or ‘excluded’ as the independent binary variable.

If we found a significant difference in values of a trait between the pool of included and excluded species, we ran additional individual predictor models that included only the life history trait in question (as well as introduction effort at the stages of establishment and spread) for all species with data on that trait to check that sampling effects did not affect our interpretation of the main result. The expectation was that important traits in multi-predictor models should also be important traits in individual predictor models that included all available data for a given variable.

1. **SUPPLEMENTARY RESULTS**
   1. **Multicollinearity: variance inflation factors (VIF) and reduced models**

In amphibians no variable has VIFs greater than five (Table S2). In reptiles however some traits have VIFs greater than five (Table S3). At each stage the variable with the highest VIF is body mass (BM). Following the protocol (section 1.2.2.) we remove BM from each stage and recalculate VIFs. The resulting VIFs are all below five indicating that are no problematic levels of multicollinearity in the reduced models.

The reduced models without BM produce qualitatively identical results to those presented in the main text. We find the same effect reported in the main text of increased clutch size, clutch frequency and reproductive lifespan on the probability of being introduced (Table S4a), increased clutch frequency on successful establishment (Table S4b), and shorter reproductive lifespan at spread (Table S4c). Heritability values are also very similar to those observed in the main models (Figure S1).

**Table S2**. Variance inflation factors (VIF) of the independent variables in the full amphibian models at each invasion stage (introduction, establishment and spread) are lower than 5, indicating that multicollinearity between predictors in not problematic. The independent variables in the table are reported as follows: introduction effort (IE), snout-vent length (SVL), egg size (ES), clutch size (CS), sexual maturity (SM), reproductive lifespan (RL).

| **VIF for:** | **Introduction** | **Establishment** | **Spread** |
| --- | --- | --- | --- |
| IE | NA | 1.09 | 1.09 |
| SVL | 1.79 | 1.73 | 3.24 |
| ES | 2.86 | 2.15 | 2.71 |
| CS | 2.50 | 2.14 | 4.96 |
| SM | 1.30 | 1.14 | 1.13 |
| RL | 1.16 | 1.35 | 1.14 |

**Table S3**. Variance inflation factors (VIF) of the independent variables in the full reptile models and the reduced models at each invasion stage (introduction, establishment and spread). Values of VIF greater than 5 are considered potentially problematic (see section 1.2.2 for details). The independent variables in the table are reported as follows: introduction effort (IE), adult body mass (BM), hatchling mass (HM), clutch size (CS), clutches per year (CY), age at sexual maturity (SM), reproductive lifespan (RL) and parity (PA). For the reduced models ‘NA’ indicates that a given independent variable has been excluded from the models due to its high VIF value in the full model.

| **Invasion stage** | **Introduction** |  | **Establishment** |  | **Spread** |  |
| --- | --- | --- | --- | --- | --- | --- |
| **VIF for:** | **Full model** | **Reduced model** | **Full model** | **Reduced model** | **Full model** | **Reduced model** |
| IE | NA | NA | 1.13 | 1.12 | 1.12 | 1.12 |
| BM | 16.85 | NA | 17.62 | NA | 13.73 | NA |
| HM | 10.09 | 2.59 | 9.95 | 2.79 | 8.46 | 3.18 |
| CS | 2.95 | 1.57 | 6.66 | 1.96 | 3.16 | 3.70 |
| CY | 1.78 | 1.36 | 4.84 | 2.01 | 2.53 | 3.14 |
| SM | 2.92 | 2.35 | 4.56 | 3.06 | 3.68 | 3.56 |
| RL | 1.94 | 1.90 | 3.06 | 2.69 | 2.90 | 2.91 |
| PA | 1.41 | 1.40 | 2.11 | 1.74 | 1.75 | 2.10 |

**Table S4.** Comparison of the reptile results between the full model (with all life history traits, as presented in the main text) and the reduced models (where adult body mass has been excluded after controlling for multicollinearity, see Table S2). For each independent variable we report the mean and SD of the *β* posterior distribution, and the percentage of *β* posterior distribution (% *β*) beyond zero (see main text for details). Stages of invasion: in (a) introduction, in (b) establishment, and in (c) spread. The independent variables in the table are reported as follows: introduction effort (IE), adult body mass (BM), hatchling mass (HM), clutch size (CS), clutches per year (CY), age at sexual maturity (SM), reproductive lifespan (RL) and parity (PA). For the reduced models ‘NA’ indicates that a given variable is not included.

| **(a) Introduction** | **Full model** | | | **Reduced model** | | |
| --- | --- | --- | --- | --- | --- | --- |
| **Statistics** | **Mean *β*** | **SD *β*** | **% *β*** | **Mean *β*** | **SD *β*** | **% *β*** |
| BM | 0.15 | 0.44 | 36.8 | NA | NA | NA |
| HM | -0.04 | 0.51 | 47.8 | 0.09 | 0.28 | 37.23 |
| CS | 1.36 | 0.58 | 1.1 | 1.44 | 0.35 | 0.03 |
| CY | 1.22 | 0.58 | 1.6 | 1.27 | 0.47 | 0.91 |
| SM | -0.41 | 0.59 | 24.4 | -0.37 | 0.55 | 24.68 |
| RL | 1.17 | 0.35 | 0.0 | 1.17 | 0.34 | 0.0 |
| PA | -0.30 | 0.38 | 21.3 | -0.29 | 0.38 | 21.91 |

| **(b) Establishment** | **Full model** | | | **Reduced model** | | |
| --- | --- | --- | --- | --- | --- | --- |
| **Statistics** | **Mean *β*** | **SD *β*** | **% *β*** | **Mean *β*** | **SD *β*** | **% *β*** |
| IE | 1.03 | 0.52 | 2.0 | 1.02 | 0.52 | 2.04 |
| BM | -0.28 | 0.84 | 36.6 | NA | NA | NA |
| HM | -0.33 | 0.76 | 32.9 | -0.56 | 0.50 | 12.13 |
| CS | 0.36 | 1.06 | 37.1 | 0.07 | 0.82 | 46.64 |
| CY | 2.11 | 1.39 | 4.5 | 1.89 | 1.21 | 3.80 |
| SM | -0.04 | 1.37 | 47.6 | -0.19 | 1.30 | 42.50 |
| RL | -1.03 | 1.05 | 15.7 | -1.04 | 0.99 | 14.02 |
| PA | 0.58 | 0.79 | 22.4 | 0.63 | 0.75 | 19.11 |

| **(c) Spread** | **Full model** | | | **Reduced model** | | |
| --- | --- | --- | --- | --- | --- | --- |
| **Statistics** | **Mean *β*** | **SD *β*** | **% *β*** | **Mean *β*** | **SD *β*** | **% *β*** |
| IE | 2.59 | 1.67 | 4.1 | 2.56 | 1.63 | 3.50 |
| BM | 1.61 | 2.22 | 23.5 | NA | NA | NA |
| HM | -1.26 | 1.85 | 24.8 | 0.39 | 1.23 | 36.57 |
| CS | 1.00 | 2.65 | 35.6 | -0.08 | 1.98 | 48.07 |
| CY | 2.78 | 2.71 | 15.0 | 1.74 | 2.21 | 20.02 |
| SM | 1.45 | 2.65 | 28.4 | 0.75 | 2.38 | 35.95 |
| RL | -3.07 | 1.94 | 4.6 | -3.25 | 1.85 | 3.04 |
| PA | -0.16 | 2.21 | 48.6 | -0.09 | 2.12 | 49.32 |


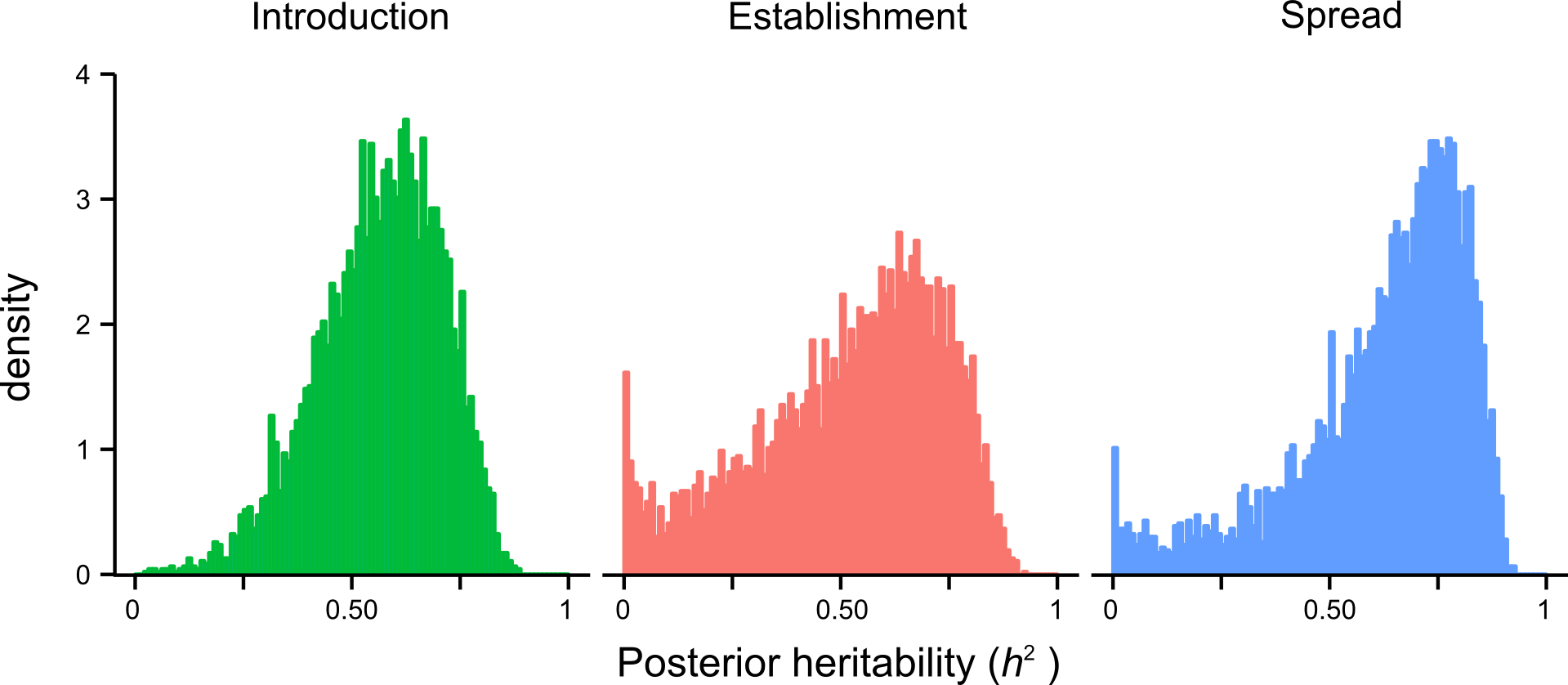


Figure S1. Posterior distribution of heritability (*h^2^*) at introduction (green), establishment (red) and spread (blue) for the reduced reptile model in which multicollinearity is resolved.

- 1. **Offspring value and life history traits**

High OV (Bókony *et al.* 2009) in reptiles is related to smaller adult and hatchling body size, less frequent clutches, earlier maturity and shorter reproductive lifespans at the stage of introduction (Table S5). The relationship with clutch frequency and reproductive lifespan persists in the smaller establishment and spread stage samples (Table S5) and at these stages a relationship with smaller clutch size is also observed. Because testing OV with other life history traits would create problems with multicollinearity of predictors, we do not include OV as an additional predictor in the main models. When analysing reptile offspring value in a reduced model with only body mass as an additional predictor (main text), we find that low offspring value is a good predictor of success at introduction but is unrelated to outcomes at establishment or spread. We suggest that the result at introduction reflects the strong relationship of OV with other, better, predictors of introduction (large and frequent clutches and long reproductive lifespan) as previously found in mammals (Capellini *et al.* 2015).

**Table S5.** Pairwise phylogenetic generalized linear models between reptile offspring value index (OV) and each life history trait (LH) at each stage of invasion (see section 1.2.3). We report the mean and SD of posterior distribution of *β* estimates for the slope of the association between OV and each life history trait, and the percentage of *β* posterior distribution crossing over 0. Variable names: IE (introduction effort), BM (adult body mass), HM (hatchling mass), CS (clutch size), CY (clutches per year), SM (age at sexual maturity), RL (reproductive lifespan) and PA (parity).

| **Stage** | **Introduction** | | | **Establishment** | |  | **Spread** | |  |
| --- | --- | --- | --- | --- | --- | --- | --- | --- | --- |
| **LH** | **Mean *β*** | **SD *β*** | **% *β*** | **Mean *β*** | **SD *β*** | **% *β*** | **Mean *β*** | **SD *β*** | **% *β*** |
| BM | -0.22 | 0.03 | 0.0 | -0.07 | 0.05 | 6.2 | -0.05 | 0.08 | 25.7 |
| HM | -0.29 | 0.04 | 0.0 | -0.06 | 0.06 | 15.4 | -0.06 | 0.10 | 28.9 |
| CS | -0.11 | 0.08 | 10.0 | 0.18 | 0.11 | 5.0 | 0.51 | 0.15 | 0.1 |
| CY | -0.69 | 0.09 | 0.0 | -0.68 | 0.12 | 0.0 | -0.77 | 0.17 | 0.0 |
| SM | -0.18 | 0.09 | 1.8 | 0.02 | 0.15 | 45.9 | 0.04 | 0.23 | 43.8 |
| RL | -0.92 | 0.03 | 0.0 | -0.85 | 0.07 | 0.0 | -0.93 | 0.10 | 0.0 |
| PA | 0.09 | 0.07 | 10.0 | 0.01 | 0.10 | 46.3 | -0.04 | 0.23 | 43.4 |

- 1. **Potential sampling effects**

We compare whether there are differences in life history traits between the ‘included’ amphibians and reptiles in the main analysis, that have all life history traits, compared to the ‘excluded’ species that are missing at least one life history trait. If present, such differences might introduce a sampling effect to our sample (section 1.1.4). Phylogenetic t-tests show that there are small differences between groups. Included amphibians have longer bodies, larger clutches, longer reproductive lifespans and a trend towards later maturity (Table S6a). Included reptiles have heavier bodies, larger and more frequent clutches (Table S6b).

**Table S6.** Phylogenetic t-test in PGLS on the mean values of each life history trait between the ‘included’ samples of amphibian (*n* = 147) and reptile (*n* = 402) species (i.e. with complete life history data) and those excluded because of incomplete life history information (‘Excluded’, section 1.2.4). The sample size of the species excluded from our analysis (‘*n* excluded’) varies for each life history trait tested depending on data availability, whereas the sample size for included species remains the same (species with complete life history information). We also report the t-value, p-value and strength of phylogenetic signal as estimated by the *λ* parameter in PGLS. Variable names: SVL (snout-vent length), ES (egg size), CS (clutch size), SM (age at sexual maturity), RL (reproductive lifespan), BM (body mass), HM (hatchling mass), CY (clutches per year).

1. **Amphibians**

| **Life history trait** | ***n* excluded** | ***β*** | **T** | **P** | ***λ*** |
| --- | --- | --- | --- | --- | --- |
| SVL | 1315 | 0.03 | 2.65 | 0.01 | 0.97 |
| ES | 691 | -0.02 | 1.22 | 0.22 | 0.96 |
| CS | 728 | 0.19 | 3.95 | <0.01 | 0.96 |
| SM | 87 | 0.05 | 1.92 | 0.06 | 0.74 |
| RL | 49 | 0.12 | 2.15 | 0.03 | 0.28 |

**(b) Reptiles**

| **Life history trait** | ***n* excluded** | ***β*** | **T** | **P** | ***λ*** |
| --- | --- | --- | --- | --- | --- |
| BM | 1557 | 0.15 | 6.39 | <0.01 | 0.98 |
| HM | 1227 | 0.02 | 1.16 | 0.25 | 0.98 |
| CS | 1244 | 0.06 | 4.85 | <0.01 | 0.93 |
| CY | 567 | 0.03 | 2.25 | 0.02 | 0.88 |
| SM | 134 | -0.01 | 0.26 | 0.80 | 0.90 |
| RL | 102 | 0.07 | 1.54 | 0.12 | 0.75 |

- 1. **Individual predictor models of invasion success**

Because we find that there are differences between the species included in the main analysis and those excluded because they lack complete life history information, we run additional models to check that these sampling effects do not affect our interpretation of the main results. For each of the life history traits that we find to be important at each invasion stage in the main analysis, we run a model of invasion outcome in the same way as models used for the main analyses, except that it includes all species with data on that individual life history trait (i.e. species with all and partial life history data; see section 1.2.4). In the models for establishment and spread we also include introduction effort as an additional predictor of invasion outcome.

The results of these models (Table S7) show that in all but two cases the life history trait of interest remains an important predictor of invasion outcome. The exceptions are that in the individual predictor model decreased sexual maturity no longer predicts amphibian introduction (28.7% of the *β* posterior distribution crossing zero) and that the association between earlier maturity and spread success is marginally less important (5.8%). Crucially, results for the stages of establishment and spread are qualitatively similar to those in the main analysis.

**Table S7.** Comparison of the important predictors from full models reported in the main analysis that include only species with all life history traits, with single predictor models that include all species with trait data (see section 1.2.4). The single life history predictor models also include introduction effort at the stages of establishment and spread. *n* total indicates the number of species included in each analysis and *n*(1) indicates the number of these species that have been successful at that stage.

| **(a) Amphibians** |  | **Full model** | | | | | **Single LH predictor model** | | | | |
| --- | --- | --- | --- | --- | --- | --- | --- | --- | --- | --- | --- |
| **Stage** | **Trait** | ***n* total** | ***n*(1)** | **Mean *b*** | **SD *b*** | **% *b*** | ***n* total** | ***n*(1)** | **Mean *b*** | **SD *b*** | **% *b*** |
| Introduction | SVL | 147 | 70 | -1.82 | 1.08 | 3.73 | 1460 | 124 | -1.81 | 0.53 | 0.01 |
|  | CS | 147 | 70 | 1.07 | 0.37 | 0.05 | 873 | 116 | 1.19 | 0.19 | 0.01 |
|  | SM | 147 | 70 | -1.67 | 1.01 | 3.88 | 232 | 87 | -0.45 | 0.78 | 28.71 |
|  | RL | 147 | 70 | 1.93 | 0.62 | 0.02 | 196 | 72 | 2.16 | 0.56 | 0.01 |
| Establishment | SVL | 58 | 41 | -3.68 | 2.32 | 4.11 | 91 | 67 | -1.16 | 0.52 | 1.09 |
|  | CS | 58 | 41 | 1.39 | 0.66 | 1.41 | 87 | 65 | 1.14 | 0.53 | 1.38 |
| Spread | SM | 36 | 11 | -5.47 | 3.19 | 2.77 | 44 | 14 | -2.61 | 1.69 | 5.82 |

| **(b) Reptiles** |  | **Full model** | | | | | **Single LH predictor model** | | | | |
| --- | --- | --- | --- | --- | --- | --- | --- | --- | --- | --- | --- |
| **Stage** | **Trait** | ***n* total** | ***n*(1)** | **Mean *b*** | **SD *b*** | **% *b*** | ***n* total** | ***n*(1)** | **Mean *b*** | **SD *b*** | **% *b*** |
| Introduction | CS | 402 | 155 | 1.36 | 0.58 | 1.1 | 1628 | 315 | 1.76 | 0.32 | 0.01 |
|  | CY | 402 | 155 | 1.22 | 0.58 | 1.6 | 957 | 262 | 1.29 | 0.35 | 0.02 |
|  | RL | 402 | 155 | 1.17 | 0.35 | 0 | 498 | 175 | 1.21 | 0.30 | 0.01 |
| Establishment | CY | 111 | 66 | 2.11 | 1.39 | 4.5 | 172 | 100 | 2.43 | 0.76 | 0.04 |
| Spread | RL | 53 | 11 | -3.07 | 1.94 | 4.6 | 56 | 11 | -2.15 | 1.29 | 3.05 |

- 1. **Alternative transformations of introduction effort**

Introduction effort measured as the number of unique locations (section 1.1.3) has a positively skewed distribution which is not normalised by any transformation. In the main analysis we convert the number of introduction locations by splitting its distribution at the median (SI 1.1.3). Here we show that the main conclusions of the study regarding the influence of life history traits on outcomes at each stage of invasion are unaltered by alternative transformations of introduction effort. We reanalyse data with the same model parameterisation but using different split points for the binary conversion of introduction effort. We also analyse the raw number of locations and log_10_ transformed number of locations.

Results in amphibians are completely consistent for different introduction effort transformations at the stage of establishment; increased introduction effort, small body size and large clutches are always important predictors of success (Table S8). At the stage of spread introduction effort is an important predictor except at the [≤4, ≥5] split, and early maturity is important except with raw introduction effort data.

In reptiles the importance of increased introduction effort on successful establishment is always found regardless of split point (Table S8b). The importance of more frequent clutches on establishment is weaker at higher splits, but stronger at the [1, ≥2] split. Short reproductive lifespan becomes a weak predictor of success at splits above [≤5, ≥6] and with raw and log transformed data, in line with results at spread. At the stage of spread the effect of introduction effort becomes unstable; at several splits it is not an important predictor ([1, ≥2] and between [≤4, ≥5] and [≤7, ≥8]). The effect of decreased reproductive lifespan is fairly consistent though results are weaker at [1, ≥2] and [≤4, ≥5] splits. These are the stages at which introduction effort is not found to be important, suggesting that it is not effectively being controlled for at these splits.

**Table S8.** Phylogenetic GLMM models of amphibian success at (a) establishment and (b) spread against all life history traits and introduction effort, when introduction effort (IE) is analysed as raw data, log-transformed data or is transformed into a binary variable using different thresholds (establishment stage: *n*=69; spread stage: *n*=36; see Figure 1a in the main text). The ‘Transform’ column indicates how IE is treated and the *n*[1] column indicates how many species falls in the category ‘1’ for IE treated as binary (*n* [1]). The median split at 2 is used in the main analysis (0 as ≤2, 1 as ≥3). The remaining columns report the mean *β* estimates and proportion of overlap with 0 for each variable in the model. Variable names: IE (introduction effort), SVL (snout-vent length), ES (egg size), CS (clutch size), SM (sexual maturity), RL (reproductive lifespan). At the 9/10 split so few species have high introduction effort it is unlikely propagule pressure is effectively controlled for here.

| **(a) Establishment** | | **IE** |  | **SVL** |  | **ES** |  | **CS** |  | **SM** |  | **RL** |  |
| --- | --- | --- | --- | --- | --- | --- | --- | --- | --- | --- | --- | --- | --- |
| **Transform** | ***n*[1]** | ***β*** | **%** | ***β*** | **%** | ***β*** | **%** | ***β*** | **%** | ***β*** | **%** | ***β*** | **%** |
| Raw data (n = 58) | NA | 2.76 | 0.0 | -3.59 | 4.8 | 1.91 | 25.1 | 1.23 | 3.3 | -0.78 | 34.2 | -0.52 | 34.0 |
| Log_10_ | NA | 2.37 | 0.7 | -4.17 | 4.2 | 4.06 | 9.0 | 1.64 | 1.0 | -2.09 | 13.3 | -1.07 | 22.5 |
| 1, ≥2 | 46 | 2.36 | 0.3 | -4.20 | 4.5 | 4.04 | 8.9 | 1.64 | 1.0 | -2.03 | 14.3 | -1.06 | 22.1 |
| Median ≤2, ≥3 | 36 | 1.29 | 3.8 | -3.68 | 4.1 | 3.48 | 10.7 | 1.39 | 1.4 | -2.08 | 11.1 | -0.75 | 26.7 |
| ≤3, ≥4 | 32 | 1.44 | 3.0 | -3.73 | 3.9 | 3.21 | 12.4 | 1.34 | 1.6 | -2.10 | 11.3 | -0.77 | 27.0 |
| ≤4, ≥5 | 29 | 1.29 | 4.1 | -3.90 | 4.2 | 3.20 | 14.1 | 1.31 | 2.8 | -1.90 | 14.4 | -0.59 | 31.9 |
| ≤5, ≥6 | 22 | 2.53 | 0.2 | -4.55 | 2.9 | 4.02 | 9.9 | 1.34 | 3.6 | -1.82 | 18.5 | -0.53 | 33.8 |
| ≤6, ≥7 | 18 | 3.09 | 0.1 | -5.28 | 1.6 | 3.98 | 11.2 | 1.34 | 4.4 | -1.61 | 22.8 | -0.36 | 38.9 |
| ≤7, ≥8 | 16 | 2.91 | 0.2 | -4.89 | 1.9 | 4.12 | 9.6 | 1.43 | 3.0 | -2.18 | 14.3 | -0.52 | 34.5 |
| ≤8, ≥9 | 14 | 2.79 | 0.2 | -4.84 | 2.5 | 4.11 | 10.9 | 1.51 | 2.2 | -2.04 | 15.3 | -0.55 | 33.0 |
| ≤9, ≥10 | 8 | 2.78 | 0.0 | -3.62 | 4.1 | 1.96 | 24.6 | 1.22 | 3.5 | -0.72 | 35.0 | -0.49 | 33.6 |

| **(b) Spread** | | **IE** |  | **SVL** |  | **ES** |  | **CS** |  | **SM** |  | **RL** |  |
| --- | --- | --- | --- | --- | --- | --- | --- | --- | --- | --- | --- | --- | --- |
| **Transform** | ***n* [1]** | ***β*** | **%** | ***β*** | **%** | ***β*** | **%** | ***β*** | **%** | ***β*** | **%** | ***β*** | **%** |
| Raw data (n = 37) | NA | 2.84 | 0.0 | -7.87 | 11.9 | 8.51 | 7.4 | 2.67 | 6.7 | -5.61 | 11.0 | -1.47 | 26.1 |
| Log_10_ | NA | 2.62 | 0.0 | -1.48 | 38.8 | 5.84 | 9.8 | 2.01 | 7.5 | -5.48 | 1.7 | 2.60 | 6.7 |
| 1, ≥2 | 31 | 2.52 | 0.0 | -1.47 | 38.5 | 5.84 | 9.4 | 2.03 | 6.8 | -5.58 | 1.4 | 2.63 | 6.6 |
| Median ≤2, ≥3 | 25 | 2.31 | 2.6 | 3.27 | 30.9 | 5.04 | 15.4 | 1.03 | 28.1 | -5.47 | 2.8 | 1.27 | 23.4 |
| ≤3, ≥4 | 24 | 2.45 | 2.0 | 3.56 | 28.6 | 4.67 | 17.8 | 0.91 | 29.8 | -5.32 | 3.5 | 1.15 | 25.0 |
| ≤4, ≥5 | 22 | 1.34 | 8.9 | -0.31 | 48.1 | 6.79 | 7.6 | 1.78 | 13.1 | -5.63 | 2.4 | 1.76 | 15.1 |
| ≤5, ≥6 | 19 | 1.81 | 4.1 | -0.51 | 45.3 | 6.51 | 8.3 | 1.57 | 15.0 | -5.27 | 3.2 | 1.65 | 16.8 |
| ≤6, ≥7 | 16 | 2.41 | 1.1 | -1.53 | 38.0 | 5.61 | 11.4 | 1.40 | 17.6 | -4.83 | 4.3 | 1.61 | 17.8 |
| ≤7, ≥8 | 14 | 2.75 | 0.9 | -3.18 | 29.3 | 7.82 | 5.7 | 2.13 | 8.7 | -6.47 | 1.8 | 1.00 | 29.1 |
| ≤8, ≥9 | 13 | 3.07 | 0.7 | -4.24 | 23.6 | 8.16 | 5.0 | 2.25 | 7.8 | -6.28 | 2.5 | 0.59 | 37.3 |
| ≤9, ≥10 | 8 | 4.05 | 0.1 | -5.36 | 20.1 | 7.61 | 8.3 | 2.48 | 7.5 | -5.99 | 3.6 | 0.34 | 42.8 |

**Table S9.** Phylogenetic GLMM models of reptile success at (a) establishment and (b) spread against all life history traits and introduction effort, when introduction effort (IE) is analysed as raw data, log-transformed data or is transformed into a binary variable using different thresholds (establishment stage: *n*=111; spread stage: *n*=53; see Figure 1a in the main text). The ‘Transform’ column indicates how IE is treated and the *n*[1] column indicates how many species falls in the category ‘1’ for IE treated as binary (*n* [1]). The median split at 2 is used in the main analysis (0 as ≤2, 1 as ≥3). The remaining columns report the mean *β* estimates and proportion of overlap with 0 for each variable in the model. Variable names: IE (introduction effort), BM (body mass), HM (hatchling mass), CS (clutch size), CY (clutches per year), SM (sexual maturity), RL (reproductive lifespan), PA (parity).

| **(a) Establishment** | | **IE** |  | **BM** |  | **HM** |  | **CS** |  | **CY** |  | **SM** |  | **RL** |  | **PA** |  |
| --- | --- | --- | --- | --- | --- | --- | --- | --- | --- | --- | --- | --- | --- | --- | --- | --- | --- |
| **Transform** | ***n* [1]** | ***β*** | **%** | ***β*** | **%** | ***β*** | ***β*** | **%** | **%** | ***β*** | **%** | ***β*** | **%** | ***β*** | **%** | ***β*** | **%** |
| Raw data (n = 155) | NA | 2.46 | 0.1 | 0.10 | 43.8 | 0.11 | 0.18 | 46.6 | 46.5 | 1.96 | 7.8 | -0.63 | 24.4 | -1.91 | 4.4 | 0.47 | 28.4 |
| Log_10_ | NA | 2.46 | 0.1 | 0.07 | 46.1 | 0.13 | 0.18 | 46.1 | 45.4 | 2.01 | 8.1 | -0.59 | 25.1 | -1.93 | 4.4 | 0.48 | 28.3 |
| 1, ≥2 | 91 | 1.88 | 0.1 | -0.72 | 16.6 | 0.89 | 0.41 | 39.5 | 20.2 | 2.72 | 1.7 | 0.07 | 46.5 | -0.99 | 16.4 | 0.38 | 30.9 |
| Median ≤2, ≥3 | 72 | 1.03 | 2.0 | -0.33 | 32.9 | 0.36 | -0.04 | 47.6 | 37.1 | 2.11 | 4.5 | -0.28 | 36.6 | -1.03 | 15.7 | 0.58 | 22.4 |
| ≤3, ≥4 | 63 | 1.22 | 0.8 | -0.17 | 41.6 | 0.22 | 0.17 | 46.1 | 42.4 | 2.11 | 5.0 | -0.41 | 31.1 | -1.23 | 12.8 | 0.56 | 23.1 |
| ≤4, ≥5 | 48 | 1.29 | 0.6 | -0.35 | 32.0 | 0.37 | 0.39 | 40.3 | 37.4 | 2.06 | 5.5 | -0.23 | 38.7 | -1.12 | 14.1 | 0.24 | 38.3 |
| ≤5, ≥6 | 37 | 2.04 | 0.1 | -0.14 | 43.6 | 0.10 | 0.65 | 34.7 | 46.9 | 2.06 | 7.1 | -0.43 | 31.8 | -1.90 | 4.9 | 0.47 | 29.1 |
| ≤6, ≥7 | 35 | 1.93 | 0.1 | -0.13 | 43.5 | 0.09 | 0.44 | 39.5 | 47.3 | 2.02 | 7.1 | -0.43 | 31.3 | -1.84 | 5.3 | 0.43 | 29.9 |
| ≤7, ≥8 | 33 | 1.79 | 0.3 | -0.19 | 41.8 | 0.18 | 0.33 | 42.0 | 44.4 | 1.98 | 7.6 | -0.41 | 32.3 | -1.71 | 6.4 | 0.35 | 33.8 |
| ≤8, ≥9 | 31 | 1.71 | 0.6 | -0.18 | 41.7 | 0.20 | 0.23 | 45.1 | 43.1 | 2.01 | 7.2 | -0.44 | 31.2 | -1.63 | 6.9 | 0.36 | 33.4 |
| ≤9, ≥10 | 27 | 2.17 | 0.1 | 0.05 | 46.3 | 0.11 | 0.05 | 49.3 | 47.8 | 1.91 | 8.5 | -0.54 | 27.6 | -1.81 | 5.5 | 0.36 | 32.6 |

| **(b) Spread** | | **IE** |  | **BM** |  | **HM** |  | **CS** |  | **CY** |  | **SM** |  | **RL** |  | **PA** |  |
| --- | --- | --- | --- | --- | --- | --- | --- | --- | --- | --- | --- | --- | --- | --- | --- | --- | --- |
| **Transform** | ***n* [1]** | ***β*** | **%** | ***β*** | **%** | ***β*** | ***β*** | **%** | **%** | ***β*** | **%** | ***β*** | **%** | ***β*** | **%** | ***β*** | **%** |
| Raw data (n = 66) | NA | 2.11 | 1.8 | 0.07 | 47.6 | -0.16 | 0.06 | 48.1 | 47.5 | 1.41 | 28.9 | 0.97 | 31.7 | -3.70 | 2.4 | -0.45 | 44.3 |
| Log_10_ | NA | 2.12 | 2.1 | 0.11 | 46.5 | -0.18 | -0.01 | 49.2 | 46.7 | 1.36 | 30.3 | 0.93 | 33.5 | -3.67 | 2.4 | -0.42 | 44.5 |
| 1, ≥2 | 49 | 0.18 | 47.3 | -1.43 | 20.2 | 1.53 | 0.71 | 37.5 | 27.2 | 2.98 | 11.2 | 1.94 | 16.0 | -2.23 | 10.0 | -0.62 | 39.3 |
| Median ≤2, ≥3 | 41 | 2.59 | 4.1 | -1.26 | 24.8 | 1.00 | 1.45 | 28.4 | 35.6 | 2.78 | 15.0 | 1.61 | 23.5 | -3.07 | 4.6 | -0.16 | 48.6 |
| ≤3, ≥4 | 37 | 2.15 | 5.3 | -1.25 | 24.8 | 0.88 | 1.81 | 24.6 | 37.0 | 2.81 | 14.1 | 1.75 | 21.8 | -3.52 | 3.5 | -0.20 | 49.3 |
| ≤4, ≥5 | 30 | -0.14 | 44.0 | -1.55 | 19.0 | 1.76 | 0.68 | 37.8 | 25.2 | 3.12 | 10.9 | 2.00 | 16.4 | -2.04 | 12.8 | -0.55 | 41.1 |
| ≤5, ≥6 | 25 | 0.98 | 16.1 | -1.04 | 28.0 | 1.10 | 0.72 | 38.1 | 33.6 | 2.43 | 17.0 | 1.73 | 18.9 | -2.87 | 6.2 | -0.52 | 42.3 |
| ≤6, ≥7 | 24 | 1.35 | 8.9 | -0.66 | 35.8 | 0.71 | 0.53 | 40.7 | 39.1 | 2.22 | 19.3 | 1.47 | 23.6 | -3.19 | 4.4 | -0.54 | 41.5 |
| ≤7, ≥8 | 23 | 1.37 | 8.5 | -0.66 | 36.2 | 0.67 | 0.57 | 39.2 | 40.5 | 2.22 | 18.4 | 1.51 | 22.1 | -3.23 | 4.4 | -0.54 | 42.1 |
| ≤8, ≥9 | 21 | 1.70 | 4.3 | -0.43 | 42.6 | 0.67 | 0.44 | 41.9 | 39.6 | 2.15 | 19.9 | 1.21 | 28.4 | -3.39 | 3.3 | -0.65 | 39.4 |
| ≤9, ≥10 | 20 | 2.03 | 2.5 | 0.08 | 47.7 | -0.23 | 0.11 | 47.3 | 45.9 | 1.49 | 28.8 | 1.02 | 31.8 | -3.74 | 2.5 | -0.65 | 39.8 |

1. **SUPPLEMENTARY DISCUSSION**

The additional analyses presented here increase confidence that that the results and conclusions in the main text are unaffected by issues of multicollinearity between predictors, sampling effects or alternative transformations of introduction effort.

Offspring value is unrelated to establishment and spread in reptiles, as found in mammals (Capellini *et al.* 2015). Additionally, offspring value is strongly associated with other life history traits; reptiles with high offspring value have smaller bodies and offspring, less frequent clutches, earlier maturity and shorter reproductive lifespans. Because clutch frequency is not commonly reported in amphibians, we cannot establish whether offspring value is bird-like or mammal/reptile-like in this class.

Multicollinearity is potentially problematic for analysis including several life history traits. However, VIFs are greater than five only in reptiles, not amphibians. High VIFs largely result from allometric relationships, particularly between hatchling mass and body mass. Once body mass is removed (leaving hatchling mass to control for allometry), there is no further problematic collinearity between the remaining predictors, and the full and reduced models are consistent in identifying the same variables as important predictors at each stage.

The differences between the species with full life history data and those with partial life history data for some traits imply that the results of the main analysis might be influenced by sampling effects. We investigate this possibility by running individual predictor models including only a single life history trait as a predictor (as well as introduction effort at establishment and spread), and including all species with data on these traits. These analyses show that in all but two cases the life history trait remains an important predictor of invasion outcome, and confirm that our conclusions are not affected by sampling effects (main text). The only exceptions are that age at sexual maturity is no longer important at the stage of introduction in amphibians, and the association between earlier maturity and success at spread becomes weaker in amphibians when all species are tested.

We are unable to replicate Mahoney et al.’s (2015) finding of an association between parthenogenesis and establishment success in reptiles because of insufficient sample size. Of 54 parthenogenetic species identified, 19 have complete life history data and are included in our analyses, 5 are introduced, 4 established and 1, *Hemidactylus garnotii,* spread. Indeed Mahoney *et al.* (2015) do not find a significant relationship between parthenogenesis and establishment success in analyses at the species level, as *Rhamphotyphlops braminus* (flowerpot snake) made up 55% of all event-level introductions of parthenogenetic species in their study.

1. **ADDITIONAL REFERENCES**
   1. **Supplementary information references**

Bauwens, D. & Diaz-Uriarte, R. (1997). Covariation of life-history traits in lacertid lizards: a comparative study. *Am. Nat.*, 149, 91–111

Blackburn, T.M., Pyšek, P., Bacher, S., Carlton, J.T., Duncan, R.P., Jarošík, V., *et al.* (2011). A proposed unified framework for biological invasions. *Trends Ecol. Evol.*, 26, 333–339

Bókony, V., Lendvai, Á.Z., Liker, A., Angelier, F., Wingfield, J.C. & Chastel, O. (2009). Stress response and the value of reproduction: are birds prudent parents? *Am. Nat.*, 173, 589–598

Bomford, M., Kraus, F., Barry, S.C. & Lawrence, E. (2009). Predicting establishment success for alien reptiles and amphibians: a role for climate matching. *Biol. Invasions*, 11, 713–724

Broennimann, O., Fitzpatrick, M.C., Pearman, P.B., Petitpierre, B., Pellissier, L., Yoccoz, N.G., *et al.* (2012). Measuring ecological niche overlap from occurrence and spatial environmental data. *Glob. Ecol. Biogeogr.*, 21, 481–497

Capellini, I., Baker, J., Allen, W.L., Street, S.E. & Venditti, C. (2015). The role of life history traits in mammalian invasion success. *Ecol. Lett.*, 18, 1099–1107

Cassey, P., Blackburn, T.M., Sol, D., Duncan, R.P. & Lockwood, J.L. (2004). Global patterns of introduction effort and establishment success in birds. *Proc. R. Soc. B Biol. Sci.*, 271, S405–S408

Clobert, J., Garland, T. & Barbault, R. (1998). The evolution of demographic tactics in lizards: a test of some hypotheses concerning life history evolution. *J. Evol. Biol.*, 11, 329–364

Colautti, R.I., Grigorovich, I.A. & MacIsaac, H.J. (2006). Propagule pressure: a null model for biological invasions. *Biol. Invasions*, 8, 1023–1037

DAISIE. (2008). *Handbook of alien species in Europe*. Springer

Duncan, R.P., Blackburn, T.M., Rossinelli, S. & Bacher, S. (2014). Quantifying invasion risk: the relationship between establishment probability and founding population size. *Methods Ecol. Evol.*, 5, 1255–1263

Duncan, R.P., Bomford, M., Forsyth, D.M. & Conibear, L. (2001). High predictability outcomes and the geographical Australian range size of introduced birds : a role for climate. *J. Anim. Ecol.*, 70, 621–632

Dunham, A.E. & Miles, D.B. (1985). Patterns of covariation in life history traits of squamate reptiles: the effects of size and phylogeny reconsidered. *Am. Nat.*, 126, 231–257

Di Febbraro, M., Lurz, P.W.W., Genovesi, P., Maiorano, L., Girardello, M. & Bertolino, S. (2013). The use of climatic niches in screening procedures for introduced species to evaluate risk of spread: a case with the American eastern grey squirrel. *PLoS One*, 8, e66559

Forsyth, D.M. & Duncan, R.P. (2001). Propagule size and the relative success of exotic ungulate and bird introductions to New Zealand. *Am. Nat.*, 157, 583–595

Forsyth, D.M., Duncan, R.P., Bomford, M. & Moore, G. (2004). Climatic suitability, life-history traits, introduction effort, and the establishment and spread of introduced mammals in Australia. *Conserv. Biol.*, 18, 557–569

Frost, D.R. (2015). *Amphibian Species of the World: an Online Reference.* Available at: http://research.amnh.org/herpetology/amphibia/index.html. Last accessed 15 March 2015

Fujisaki, I., Hart, K.M., Mazzotti, F.J., Rice, K.G., Snow, S. & Rochford, M. (2010). Risk assessment of potential invasiveness of exotic reptiles imported to South Florida. *Biol. Invasions*, 12, 2585–2596

García-Díaz, P., Ross, J. V, Ayres, C. & Cassey, P. (2015). Understanding the biological invasion risk posed by the global wildlife trade: propagule pressure drives the introduction and establishment of Nearctic turtles. *Glob. Chang. Biol.*, 21, 1078–1091

Greene, W.H. (2012). *Econometric Analysis.* 7th edn. Pearson Educations, Harlow

Hadfield, J.D. & Nakagawa, S. (2010). General quantitative genetic methods for comparative biology: Phylogenies, taxonomies and multi-trait models for continuous and categorical characters. *J. Evol. Biol.*, 23, 494–508

Invasive Species Specialist Group ISSG. (2014). *The Global Invasive Species Database. Version 2014.1*. Available at: http://www.issg.org/database.

Ives, A.R., Midford, P.E. & Garland, T. (2007). Within-species variation and measurement error in phylogenetic comparative methods. *Syst. Biol.*, 56, 252–70

Jaffe, A.L., Slater, G.J. & Alfaro, M.E. (2011). The evolution of island gigantism and body size variation in tortoises and turtles. *Biol. Lett.*, 7, 558–561

Jeschke, J.M. & Strayer, D.L. (2008). Are threat status and invasion success two sides of the same coin? *Ecography*, 31, 124–130

van Kleunen, M., Dawson, W., Schlaepfer, D., Jeschke, J.M. & Fischer, M. (2010a). Are invaders different? A conceptual framework of comparative approaches for assessing determinants of invasiveness. *Ecol. Lett.*, 13, 947–958

van Kleunen, M., Weber, E. & Fischer, M. (2010b). A meta-analysis of trait differences between invasive and non-invasive plant species. *Ecol. Lett.*, 13, 235–245

Kraus, F. (2009). *Alien Reptiles and Amphibians: A Scientific Compendium and Analysis Series*. Springer Verlag, Dordrecht, Holland

Křivánek, M., Pyšek, P. & Jarošík, V. (2006). Planting history and propagule pressure as predictors of invasion by woody species in a temperate region. *Conserv. Biol.*, 20, 1487–1498

Lever. (2003). *Naturalized Reptiles and Amphibians of the World*. Oxford University Press, New York

31.Li, Y., Liu, X., Li, X., Petitpierre, B. & Guisan, A. (2014). Residence time, expansion toward the equator in the invaded range and native range size matter to climatic niche shifts in non-native species. *Glob. Ecol. Biogeogr.*, 23, 1094–1104

Liu, X., Li, X., Liu, Z., Tingley, R., Kraus, F., Guo, Z., *et al.* (2014). Congener diversity, topographic heterogeneity and human-assisted dispersal predict spread rates of alien herpetofauna at a global scale. *Ecol. Lett.*, 17, 821–829

Lockwood, J.L., Cassey, P. & Blackburn, T. (2005). The role of propagule pressure in explaining species invasions. *Trends Ecol. Evol.*, 20, 223–228

Long, J.L. (1997). *Regression Models for Categorical and Limited Dependent Variables*. SAGE Publications Inc., London

Mahoney, P.J., Beard, K.H., Durso, A.M., Tallian, A.G., Long, A.L., Kindermann, R.J., *et al.* (2015). Introduction effort, climate matching and species traits as predictors of global establishment success in non-native reptiles. *Divers. Distrib.*, 21, 64–74

Marchetti, M.P., Moyle, P.B. & Levine, R. (2004). Invasive species profiling? Exploring the characteristics of non-native fishes across invasion stages in California. *Freshw. Biol.*, 49, 646–661

Oaks, J.R. (2011). A time-calibrated species tree of crocodylia reveals a recent radiation of the true crocodiles. *Evolution*, 65, 3285–3297

Organ, C.L., Shedlock, A.M., Meade, A., Pagel, M. & Edwards, S. V. (2007). Origin of avian genome size and structure in non-avian dinosaurs. *Nature*, 446, 180–184

Orme, D. (2013). The caper package: comparative analysis of phylogenetics and evolution in R. *R Packag. version*

Pyron, R.A., Burbrink, F.T. & Wiens, J.J. (2013). A phylogeny and revised classification of Squamata, including 4161 species of lizards and snakes. *BMC Evol. Biol.*, 13, 93

Pyron, R.A. & Wiens, J.J. (2011). A large-scale phylogeny of Amphibia including over 2800 species, and a revised classification of extant frogs, salamanders, and caecilians. *Mol. Phylogenet. Evol.*, 61, 543–583

Quinn, G. & Keough, M. (2007). *Experimental Design and Data Analysis for Biologists.* 6th edn. Cambridge University Press, Cambridge, UK

Rago, A., While, G.M. & Uller, T. (2012). Introduction pathway and climate trump ecology and life history as predictors of establishment success in alien frogs and toads. *Ecol. Evol.*, 2, 1437–1445

Revell, L.J. & Reynolds, G.R. (2012). A new Bayesian method for fitting evolutionary models to comparative data with intraspecific variation. *Evolution*, 66, 2697–2707

Richardson, D.M. & Pyšek, P. (2008). Fifty years of invasion ecology - the legacy of Charles Elton. *Divers. Distrib.*, 14, 161–168

Richardson, D.M. & Pyšek, P. (2012). Naturalization of introduced plants: ecological drivers of biogeographical patterns. *New Phytol.*, 196, 383–396

Shedlock, A.M. & Edwards, S.V. (2009). Amniotes. In: *The Timetree of Life* (eds. Hedges, S.B. & Kumar, S.). Oxford University Press, Oxford, pp. 375–379

Simberloff, D. (2009). The Role of Propagule Pressure in Biological Invasions. *Annu. Rev. Ecol. Evol. Syst.*, 40, 81–102

Sol, D., Maspons, J., Vall-llosera, M., Bartomeus, I., Garcia-Pena, G.E., Pinol, J., *et al.* (2012). Unraveling the Life History of Successful Invaders. *Science (80-. ).*, 337, 580–583

Tingley, R., Phillips, B.L. & Shine, R. (2011). Establishment success of introduced amphibians increases in the presence of congeneric species. *Am. Nat.*, 177, 382–8

Tingley, R., Thompson, M.B., Hartley, S. & Chapple, D.G. (2016). Patterns of niche filling and expansion across the invaded ranges of an Australian lizard. *Ecography (Cop.).*, 39, 270–280

Uetz, P. & Hošek, J. (2015). *The Reptile Database*. Available at: http://www.reptile-database.org.

van Wilgen, N.J. & Richardson, D.M. (2012). The Roles of Climate, Phylogenetic Relatedness, Introduction Effort, and Reproductive Traits in the Establishment of Non-Native Reptiles and Amphibians. *Conserv. Biol.*, 26, 267–277

- 1. **Data references: Biological invasions database**

Alfonso, Y.U., Casenave-Cambet, A.C., Fong, A. & Díaz, L.M. (2012). First record of the unisexual lizard *Gymnophthalmus underwoodi* (Squamata: Gymnophthalmidae) in Cuba. *Reptil. Amphib. Conserv. Nat. Hist.,* 19, 57–59.

Avery, M.L., Tillman, E.A., Spurfeld, C., Engeman, R.M., Maciejewski, K.P., Brown, J.D., et al. (2014). Invasive black spiny‐tailed iguanas (Ctenosaura similis) on Gasparilla Island, Florida, USA. *Integr. Zool*., 9, 590–597.

Bellati, A., Razzetti, E., Resteghini, M., Sacchi, R., Pellitteri-Rosa, D., Casiraghi, M., et al. (2012). First molecular characterization of invasive alien populations of Pelophylax kurtmuelleri (Gayda, 1940) and new records from Italy. In*: Atti IX Congr. Naz. della Soc. Herpetol*. Ital. pp. 287–288.

Berg, C.S., Jeremiah, A., Harrison, B. & Henderson, R.W. (2009). New island records for Tantilla melanocephala (Squamata: Colubridae) on the Grenada Bank. *Appl. Herpetol*., 6, 403.

Breuil, M. (2009). The terrestrial herpetofauna of Martinique: past, present, future. *Appl. Herpetol.*, 6, 123–149.

Buckland, S., Cole, N.C., Aguirre-Gutiérrez, J., Gallagher, L.E., Henshaw, S.M., Besnard, A., et al. (2014). Ecological effects of the invasive giant Madagascar day gecko on endemic Mauritian geckos: Applications of binomial-mixture and species distribution models. *PLoS One*, 9, e88798.

California Herps (accessed Dec 2014). URL http://californiaherps.com.

Edwards, T., Bonine, K.E., Ivanyi, C. & Prescott, R. (2005). The molecular origins of spiny-tailed iguanas (Ctenosaura) on the grounds of the Arizona-Sonora Desert Museum. *Son. Herpetol*., 18, 122–125.

Enge, K.M., Krysko, K.L., Hankins, K.R., Campbell, T.S. & King, F.W. (2004). Status of the Nile monitor (*Varanus niloticus*) in southwestern Florida. *Southeast. Nat*., 3, 571–582.

Florida Fish Wildl. Conserv. Comm. (accessed Dec 2014). URL http://myfwc.com/wildlifehabitats/nonnatives/.

Goldberg, S.R., Bursey, C.R., Sullivan, K.O., Bowker, R.W. & Sullivan, B.K. (2015). Old-World nematodes in the ocellated skink, *Chalcides ocellatus* (Squamata: Scincidae) now established in Mesa, Maricopa County, Arizona, USA. *Comp. Parasitol*., 82, 304–305.

Invasive Species of Japan (accessed Dec 2014). URL http://www.nies.go.jp/biodiversity/invasive/.

IUCN Red List (accessed Dec 2014). URL http://www.iucnredlist.org/.

King, F.W. & Krakauer, T. (1966). The exotic herpetofauna of southeastern Florida. *Quart. J. Fla. Acad. Sci.*, 29, 144-154.

Kosuch, J., Vences, M., Dubois, A., Ohler, A. & Böhme, W. (2001). Out of Asia: mitochondrial DNA evidence for an oriental origin of tiger frogs, genus Hoplobatrachus. *Mol. Phylogenet. Evol.*, 21, 398–407.

Krysko, K.L., Burgess, J., Rochford, M., Gillette, C., Cueva, D., Enge, K., et al. (2011). Verified non-indigenous amphibians and reptiles in Florida from 1863 through 2010: Outlining the invasion process and identifying invasion pathways and stages. *Zootaxa*, 3028, 1–64.

Meilink, W.R.M., Clegg, J.R., Mayerl, C.J., Pinto, J.S., Grasso, D., Stegen, G., et al. (2013). Confirmation of the presence of the sphaerodactylid lizard *Gonatodes vittatus* in Guyana, and an indication of a reproductively active population in Georgetown. *Herpetologie Terrarienkunde*

Mozzi, R., Deso, G. & Probst, J.-M. (2005). Un nouveau gecko vert introduit à La Réunion le *Phelsuma astriata semicarinata* (Cheke, 1982). *Bull. Phaethon*, 21, 1–4.

Ota, H., Toda, M., Masunaga, G., Kikukawa, A. & Toda, M. (2004). Feral populations of amphibians and reptiles in the Ryukyu Archipelago, Japan. *Glob. Environ. Res*., 8, 133–143.

Pinya, S. & Carretero, M.A. (2011). The Balearic herpetofauna: a species update and a review on the evidence. *Acta Herpetol.*, 6, 59–80.

Sanchez, M., Probst, J.-M. & Réunion, F. (2014). Distribution and habitat of the invasive giant day gecko *Phelsuma grandis* Gray 1870 (Sauria: Gekkonidae) in Reunion Island, and conservation implication. *Phelsuma*, 22, 13–28.

Scantlebury, D., Ng, J., Landestoy, M. & Glor, R.E. (2010). *Hemidactylus frenatus* and *Gymnophthalmus underwoodi* in the Dominican Republic. *Reptil. Amphib*., 17, 180–181.

da Silva Rocha, I.R. (2012). Patterns of biological invasion in the herpetofauna of the Balearic Islands: determining the origin and predicting the expansion as conservation tools. Dissertation: Universidade do Porto.

- 1. **Data references: Life history databases**

Bielby, J., Cooper, N., Cunningham, A. A., Garner, T. W. J., & Purvis, A. (2008). Predicting susceptibility to future declines in the world’s frogs. *Conservation Letters*, 1(2), 82–90.

Brown, J.L., Morales, V. & Summers, K. (2010). A key ecological trait drove the evolution of biparental care and monogamy in an amphibian. *Am. Nat*., 175, 436–46.

Byrne, P.G., Roberts, J.D. & Simmons, L.W. (2002). Sperm competition selects for increased testes mass in Australian frogs. *J. Evol. Biol*., 15, 347–355.

Carey, J.R. & Judge, D.S. (2000). Longevity records: life spans of mammals, birds, amphibians, reptiles, and fish. *Monographs on Population Aging*, 8, Odense University Press

Cooper, N., Bielby, J., Thomas, G.H. & Purvis, A. (2008). Macroecology and extinction risk correlates of frogs. *Glob. Ecol. Biogeogr*., 17, 211–221.

Feldman, A. & Meiri, S. (2013). Length-mass allometry in snakes. *Biol. J. Linn. Soc*., 108, 161–172.

Foufopoulos, J. & Ives, A.R. (1999). Reptile Extinctions on Land‐Bridge Islands: Life‐History Attributes and Vulnerability to Extinction. *Am. Nat.,* 153, 1–25.

García-Díaz, P., Ross, J. V, Ayres, C. & Cassey, P. (2015). Understanding the biological invasion risk posed by the global wildlife trade: propagule pressure drives the introduction and establishment of Nearctic turtles. *Glob. Chang. Biol.*, 21, 1078–1091.

Gomez-Mestre, I., Pyron, R.A. & Wiens, J.J. (2012). Phylogenetic analyses reveal unexpected patterns in the evolution of reproductive modes in frogs. *Evolution*, 66, 3687–700.

Grimm, A., Prieto Ramírez, A.M., Moulherat, S., Reynaud, J. & Henle, K. (2014). Life-history trait database of European reptile species. *Nat. Conserv*., 9, 45–67.

Halámková, L., Schulte, J.A. & Langen, T.A. (2013). Patterns of sexual size dimorphism in Chelonia. *Biol. J. Linn. Soc*., 108, 396–413.

Han, X. & Fu, J. (2013). Does life history shape sexual size dimorphism in anurans? A comparative analysis. *BMC Evol. Biol.*, 13, 27.

Hendry, C.R., Guiher, T.J. & Pyron, R.A. (2014). Ecological divergence and sexual selection drive sexual size dimorphism in New World pitvipers (Serpentes: Viperidae). *J. Evol. Biol*., 27, 760–71.

Hoverman, J.T., Gray, M.J., Haislip, N.A. & Miller, D.L. (2011). Phylogeny, life history, and ecology contribute to differences in amphibian susceptibility to ranaviruses. *Ecohealth*, 8, 301–19.

Lambert, S.M. & Wiens, J.J. (2013). Evolution of viviparity: a phylogenetic test of the cold-climate hypothesis in phrynosomatid lizards. *Evolution*, 67, 2614–30.

De Lisle, S.P. & Rowe, L. (2013). Correlated evolution of allometry and sexual dimorphism across higher taxa. *Am. Nat*., 182, 630–9.

De Magalhães, J.P. & Costa, J. (2009). A database of vertebrate longevity records and their relation to other life-history traits*. J. Evol. Biol*., 22, 1770–4.

Mahoney, P.J., Beard, K.H., Durso, A.M., Tallian, A.G., Long, A.L., Kindermann, R.J., et al. (2015). Introduction effort, climate matching and species traits as predictors of global establishment success in non-native reptiles. *Divers. Distrib*., 21, 64-74.

Meiri, S. (2008). Evolution and ecology of lizard body sizes. *Glob. Ecol. Biogeogr.*, 17, 724–734.

Meiri, S. (2010). Length-weight allometries in lizards. *J. Zool*., 281, 218-226

Meiri, S., Bauer, A.M., Chirio, L., Colli, G.R., Das, I., Doan, T.M., et al. (2013). Are lizards feeling the heat? A tale of ecology and evolution under two temperatures. *Glob. Ecol. Biogeogr*., 22, 834–845.

Meiri, S., Brown, J.H. & Sibly, R.M. (2012). The ecology of lizard reproductive output. *Glob. Ecol. Biogeogr.*, 21, 592–602.

Meiri, S., Feldman, A. & Kratochvíl, L. (2014). Squamate hatchling size and the evolutionary causes of negative offspring size allometry. *J. Evol. Biol*., 28(2), 438-446.

Mesquita, D.O., Colli, G.R., Costa, G.C., Costa, T.B., Shepard, D.B., Vitt, L.J., et al. (2015). Life history data of lizards of the world. *Ecology*, 96, 594–594.

Myhrvold, N.P., Baldridge, E., Chan, B., Sivam, D., Freeman, D.L. & Ernest, S.K.M. (2015). An amniote life-history database to perform comparative analyses with birds, mammals, and reptiles. *Ecological Archives*, E096-269.

Nali, R.C., Zamudio, K.R., Haddad, C.F.B. & Prado, C.P.A. (2014). Size-dependent selective mechanisms on males and females and the evolution of sexual size dimorphism in frogs. *Am. Nat.*, 184, 727–40.

Novosolov, M., Raia, P. & Meiri, S. (2013). The island syndrome in lizards. *Glob. Ecol. Biogeogr*., 22, 184–191.

Pincheira-Donoso, D., Tregenza, T., Witt, M.J. & Hodgson, D.J. (2013). The evolution of viviparity opens opportunities for lizard radiation but drives it into a climatic cul-de-sac. *Glob. Ecol. Biogeogr*., 22, 857–867.

Pyron, R.A. & Burbrink, F.T. (2014). Early origin of viviparity and multiple reversions to oviparity in squamate reptiles. *Ecol. Lett*., 17, 13–21.

Scharf, I., Feldman, A., Novosolov, M., Pincheira-Donoso, D., Das, I., Böhm, M., et al. (2015). Late bloomers and baby boomers: ecological drivers of longevity in squamates and the tuatara. *Glob. Ecol. Biogeogr*., 24, 396–405.

Scharf, I. & Meiri, S. (2013). Sexual dimorphism of heads and abdomens: Different approaches to “being large” in female and male lizards. *Biol. J. Linn. Soc*., 110, 665–673.

Sodhi, N.S., Bickford, D., Diesmos, A.C., Lee, T.M., Koh, L.P., Brook, B.W., et al. (2008). Measuring the meltdown: Drivers of global amphibian extinction and decline. *PLoS One*, 3, e1636.

Summers, K., Sea McKeon, C. & Heying, H. (2006). The evolution of parental care and egg size: a comparative analysis in frogs. *Proc. Biol. Sci*., 273, 687–92.

Thorbjarnarson, J.B. (1996). Reproductive characteristics of the order Crocodylia. *Herpetologica*, 52, 8–24.

Trochet, A., Moulherat, S., Calvez, O., Stevens, V., Clobert, J. & Schmeller, D. (2014). A database of life-history traits of European amphibians*. Biodivers. Data J.,* 2, e4123.

Van Wilgen, N.J. & Richardson, D.M. (2012). The Roles of Climate, Phylogenetic Relatedness, Introduction Effort, and Reproductive Traits in the Establishment of Non-Native Reptiles and Amphibians. *Conserv. Biol*., 26, 267–277.

Zhang, L. & Lu, Xi. (2012). Amphibians live longer at higher altitudes but not at higher latitudes. *Biol. J. Linn. Soc*., 106, 623–632.
